# Soil–Plant Transfer and Environmental Levels of Potentially Toxic Elements in Agricultural, Urban and Industrial Areas of the València Region (Eastern Spain)

**DOI:** 10.3390/toxics14050353

**Published:** 2026-04-22

**Authors:** Eva Fernández-Gómez, Luis Roca-Pérez, Jaume Bech, José Antonio Rodríguez-Martín, Rafael Boluda

**Affiliations:** 1 Plant Biology Department, Faculty of Pharmacy, Universitat de València, Av Vicent Andrés i Estellés 22, Burjassot, 46100 València, Spain; eva.fernandez-gomez@uv.es (E.F.-G.); luis.roca@uv.es (L.R.-P.); 2 Faculty of Biology, Universitat de Barcelona, Av Diagonal 643, 08023 Barcelona, Spain; jaumebechborras@gmail.com; 3 Department of Environment, Instituto Nacional de Investigación y Tecnología Agraría y Alimentaria (INIA), 28040 Madrid, Spain; rmartin@inia.csic.es

**Keywords:** major elements, heavy metals, Mediterranean calcareous soils, contamination sources, contamination indices, bioaccumulation factor

## Abstract

The evaluation of potentially toxic element concentrations (PTEs) in soils and plants is essential for understanding environmental quality and potential human exposure in areas affected by intense anthropogenic activity. This study addresses a research gap in the Valencian Region, focusing on soil–plant interactions of PTEs in urban and industrial environments. We assess the status of the soil–plant system in a region of the Valencian Community (eastern Spain) subjected to strong urban, industrial and agricultural pressure. A total of 55 soil samples and 47 plant samples were collected from agricultural, urban and industrial sites and analysed for soil properties, major elements (Al, Mg, Fe) and PTEs (As, Cd, Co, Cr, Cu, Li, Mn, Ni, Sr, V and Zn). Land use significantly influenced soil physicochemical characteristics, with clear differentiation among environments. Soil texture and organic matter were the main factors controlling element retention, while Al, Fe and Mg dominated the geochemical composition, consistent with Mediterranean calcareous soils. Correlation analyses revealed strong co-occurrence patterns among lithogenic elements (e.g., Fe-Al, r = 0.917 *p* < 0.01), soil texture and chemical properties, indicating a shared origin and preferential retention in the fine fraction and soil organic matter. Contamination indices identified potential environmental risk mainly associated with Cu, Pb, Sr and Zn, particularly in densely populated areas. Mean concentrations of Cd, Cr, Cu, Pb and Zn were, respectively, 0.63 mg kg^−1^, 42.25 mg kg^−1^, 31.49 mg kg^−1^, 56.91 mg kg^−1^ and 76.08 mg kg^−1^. These elements exceeded Spanish regulatory reference values in several soils. Bioaccumulation indices indicated notable plant uptake of As, Sr and Zn, highlighting their potential for trophic transfer.

## 1. Introduction

Soil is a natural body and a key component of the Critical Zone, forming the interface between the lithosphere, biosphere and hydrosphere and supporting essential ecosystem functions [1]. With the global population projected to reach 9.8 billion by 2050 [2], the capacity of soils to sustain food production is increasingly important, as approximately 95% of food is produced directly or indirectly through soil processes [3,4]. At the same time, soils act as reservoirs for potentially toxic elements (PTEs), which may pose risks to ecosystems and human health and contribute to the contamination of aquatic systems [5,6]. The presence of PTEs in soils originates from both natural sources—such as parent material, weathering and volcanic activity—and anthropogenic inputs, including industrial emissions, agricultural practices, waste disposal and urban activities [6,7,8,9,10,11,12,13,14]. These contributions have intensified environmental pollution worldwide, with significant implications for food security, biodiversity and public health [15].

In the Iberian Peninsula, numerous studies have documented PTE contamination in agricultural soils (e.g., [5,8,11,16,17,18,19,20,21,22,23,24]), showing soil–plant relationships for elements such as Cd, Co, Cu, Ni and Pb and demonstrating that contamination by As, Cd, Co, Cr, Cu, Hg, Ni, Pb and Zn increases significantly due to intensive agrochemical use, affecting soil fertility [25]. Industrial [5,9,26,27] and urban [28,29,30,31,32,33,34,35,36,37,38,39,40,41,42,43] soils have also received considerable attention, as commercial and industrial activities act as local sources of contamination, promoting the dispersion of PTEs and their potential transfer to plants. Urban soils are increasingly used as indicators of environmental quality [11,24,39,44,45,46,47,48], as they are strongly influenced by vehicular traffic, atmospheric deposition and the degradation of construction materials [49,50]; therefore, it is necessary to monitor this type of pollution in urban soils.

Plants play an important role in the soil–plant system, absorbing essential micronutrients (e.g., Fe, Mn, Co, Cr, Cu, Ni, Zn) required for metabolic processes [10] but also accumulating non-essential and potentially toxic elements, such as As, Cd, Hg and Pb. These elements can cause adverse health effects even at low concentrations through ingestion, inhalation or dermal absorption, including neurodegenerative disorders, musculoskeletal diseases and reproductive dysfunctions [6,10,34,51]. Many of these elements are included in the priority list of hazardous substances of the Agency for Toxic Substances and Disease Registry due to their toxicity and potential for human exposure [52]. Other elements, such as Sr and V, have recently gained attention due to their increasing environmental presence and potential toxicity [11,53,54]. Vanadium is widely used in industrial alloys and, although it may enhance certain plant physiological processes at low concentrations, its accumulation in soils and transfer to plants poses risks due to its toxic and potentially carcinogenic effects [55,56]. Strontium, commonly associated with carbonate sediments, is easily mobilised during weathering and has been linked to pollution in agricultural and industrial soils [11,57,58]. To our knowledge, there is a lack of studies examining the relationship between PTE concentrations in soils and plants in urban and industrial environments.

Soil contamination by PTEs is commonly assessed using regulatory thresholds and contamination indices, which provide a comparative evaluation of pollution levels. These include the zinc equivalent, geoaccumulation index, enrichment factor, contamination factor, pollution load index and modified degree of contamination [8,22,28,30,31,32,37,59,60,61]. Their application requires reliable background levels (C_BL_) to distinguish natural concentrations from anthropogenic enrichment [62,63], following criteria such as those established in ISO 19258 [64]. In this regard, it is important to note that background levels for the studied elements have not yet been officially established in the Valencian Community, representing a significant limitation for soil contamination assessment and risk evaluation in the region.

Given all the aforementioned aspects, the aims of this study were to: (i) determine soil properties and concentrations of major elements (Al, Fe, Mg) and PTEs (As, Cd, Co, Cr, Cu, Li, Mn, Ni, Pb, Sr, V and Zn) in topsoils and plants from agricultural, urban and industrial areas of 17 municipalities in the Valencian Region (eastern Spain); (ii) analyse the relationships between soil properties, element concentrations and enrichment indices; (iii) assess contamination patterns using pollution indices to identify spatial trends; and (iv) evaluate soil–plant transfer through bioaccumulation factors. We hypothesised that land use associated with human activities significantly influences soil characteristics and the concentration and distribution of PTEs in the soil–plant system.

## 2. Materials and Methods

### 2.1. Study Area and Sampling

The study area is located in eastern Spain, in the province of Valencia, which has a population of 2,605,757 inhabitants and covers 10,807 km^2^ [65]. Although agriculture was historically the main economic activity, currently, 67% of the regional economy is based on trade and services [24]. The region has a Mediterranean climate, with a mean annual temperature of 16 °C and annual precipitation of 527.9 mm in 2021 [66]. Soils develop on calcareous alluvial–colluvial materials, mainly classified as Fluvisols and Calcisols [67]. The study area includes the city of València (the third-largest city in Spain), 17 surrounding municipalities (Table S1), and the Albufera Natural Park (ANP), one of the few Spanish wetlands protected at the European level since 1986. Two-thirds of the ANP’s 21,000 ha are occupied by rice fields, which maintain the wetland ecosystem [19,24,68]. The ANP is bordered by extensive vegetable and citrus croplands, as well as urban and industrial areas. It is important to note that the experimental work was conducted before the major flooding event of 29 October 2024, which severely affected many of the municipalities included in this study. This provides an added value for future research assessing post-flood soil alterations.

A simple random sampling design was applied at 55 sites across the main municipalities (Table S1), yielding 55 soil samples and 47 plant samples. Sites were selected according to land use: agricultural, urban, and industrial. Between 4 and 6 subsamples were collected at each site from 0 to 20 cm depth using a Teflon-coated hand auger. Subsamples were homogenised manually with nitrile gloves, sieved to 2 mm, and stored in polyethylene bags for laboratory processing [69]. Plant material from tree, shrub, and herbaceous species was collected at each site. For trees and shrubs, mature leaves in good condition were sampled from the outer and terminal parts of branches, covering the four cardinal orientations. Entire plants were collected for herbaceous species. Samples were stored in polyethylene bags inside a portable cooler at 4–6 °C. The sampling distribution was as follows: agricultural soils (A) (S1–2, S5, S9, S13, S16–18, S22–27, S29–31, S45–51), urban parks (UPs) (S4, S6, S8, S10–11, S19–20, S32–43, S52–55), and industrial parks (IPs) (S3, S7, S12, S14–15, S28). One forest soil sample from La Devesa (ANP) was also included (S21).

In the laboratory, all samples were oven-dried at 40–50 °C. A fraction of each soil sample was ground in an agate mortar for element (ET) determination. Plant material was also dried and ground prior to analysis.

### 2.2. Analytical Methods

#### 2.2.1. Soil Characteristics

Distilled–deionised water and analytical-grade reagents were used throughout. The following soil properties were analysed, as they strongly influence the behaviour of PTEs: particle size distribution, pH, electrical conductivity (EC), carbonates, soil organic matter (SOM), total nitrogen (N), and cation exchange capacity (CEC). Particle size distribution was determined using the Bouyoucos hydrometer method [70]. Soil pH was measured in a 1:2.5 soil:water suspension using a GLP Crison pH meter (Crison, Barcelona, Spain) [71]. EC was measured in a 1:5 water extract using a PC 2700 conductimeter (EUTECH Instruments, Singapore), UNE-EN (2001) [72]. Carbonates were quantified by the gas volumetric method using Bernard’s calcimeter after adding excess 6 M HCl, following ISO 10693 [73]. SOM was estimated using Equation (1), after determining oxidisable organic carbon (Cox) by dichromate oxidation and titration with ammonium ferrous sulphate [74].

SOM = 1.72 × 1.29 × %Cox(1)

Total N was measured using an EA 1110 CNHS elemental analyser (CE Instruments, Milan, Italy) at ≥900 °C in the presence of oxygen, following ISO 13878 [75]. CEC was determined using the NaAc/NH_4_Ac method [71], and Na concentration in the final extract was measured by flame AAS (Agilent Technologies 200 Series AA, Santa Clara, CA, USA). All samples were analysed in duplicate, and results were accepted when the coefficient of variation (CV) was ≤5% except for CEC (CV ≤ 10%).

#### 2.2.2. Soil and Plant Digestion

All elements (ETs) in both matrices were extracted using acid-assisted microwave digestion. A 0.5 g of pulverised sample was weighed into a Teflon vessel to which 9 mL of nitric acid (68% *w*/*v*), 1 mL of hydrogen peroxide (33% *w*/*v*), and 3 mL of hydrochloric acid (37% *w*/*v*) were added (all from Scharlab, Barcelona, Spain). Digestion proceeded for 20 min at 200 °C in a microwave oven (Mars 6, CEM Corporation, Matthews, NC, USA [76]. ET concentrations (Al, As, Cd, Co, Cr, Cu, Fe, Li, Mg, Mn, Ni, Pb, Sr, V and Zn) in the soil and plant extracts were determined using an Inductively Coupled Plasma Optical Emission Spectrometer (ICP-OES), Thermo iCAP 6500 Duo, Thermo Fisher Scientific, Waltham, MA, USA.

Quality assurance included analysis of certified reference materials (soil: EnviroMAT SS-1, 140-025-001, SCP SCIENCE, Baie-D’Urfé, QC, Canada; plant: Citrus leaves NCS ZC73018, NACIS, Beijing, China), along with two blanks per batch. Calibration curves showed good linearity (R^2^ ≥ 0.99). Recoveries were considered acceptable within ±25% of certified values due to matrix complexity [77]. The recovery percentages obtained for soils were: Al 125.34 ± 6.82, As 87.28 ± 2.99, Cd 125.47 ± 6.46, Co 90.70 ± 11.75, Cr 121.68 ± 1.74, Cu 93.76 ± 5.23, Fe 107.24 ± 4.99, Li 125.33 ± 1.81, Mg 96.54 ± 0.52, Mn 97.95 ± 7.34, Ni 85.10 ± 10.73, Pb 100.05 ± 2.46, Sr 95.53 ± 11.02, V 125.15 ± 2.85, and Zn 101.16 ± 4.57. In the case of plant material, the recovery percentages were: Al 76.94 ± 6.02, As 80.95 ± 9.40, Cd 107.03 ± 7.56, Co 124.94 ± 2.05, Cr 102.71 ± 9.39, Cu 92.98 ± 5.96, Fe 81.94 ± 8.17, Li 122.67 ± 14.32, Mg 87.43 ± 4.47, Mn 123.22 ± 5.93, Ni 93.22 ± 12.74, Pb 95.43 ± 3.79, Sr 85.73 ± 7.62, V 79.77 ± 5.71, and Zn 100.19 ± 11.88. All results are expressed on a dry weight basis. Duplicate analyses were accepted when CV < 15%. The highest variability in soils occurred for Co and Ni (13%) and Sr (12%); the rest of the elements showed a variation range between 1% and 7%; the highest variability in plants occurred for As, Li, Zn (12%) and Ni (14%), while the rest of the elements showed a variation range between 1% and 10%. The quantification limits (LOQs) (mg kg^−1^) were: 2 for Al and Fe, and 0.01 for all other elements.

### 2.3. Soil Contamination Indices

Several indices were applied to assess soil contamination. The zinc equivalent index (ZnEq) was calculated using Equation (2), considering the combined toxicity of Zn, Cu, and Ni [8,22,78]:

ZnEq = [Zn] + 2 × [Cu] + 8 × [Ni](2)

Given the background levels (C_BL_) obtained in this study (Table 1), ZnEq > 300 mg kg^−1^ indicates potential toxicity, slightly above the 250 mg kg^−1^ threshold proposed by Chumbley [78].

Additional indices (igeo-accumulation index, Igeo, Equation (3); enrichment factor, EF, Equation (4); contamination factor, CF, Equation (5); pollution load index, PLI, Equation (6); and modified contamination degree index, mCd, Equation (7)) were calculated following [8,22,28,30,31,32,37,59,60,61]. Igeo was applied to characterise soil contamination levels, which consist of seven classes according to Bhuiyan et al. [79] and Tamim et al. [60] (Table S2). EF was used to identify the contribution of anthropogenic sources to the topsoil. Fe was used as the reference element for EF due to its geochemical stability and uniform natural distribution [59,60,61]. The PLI was used to assess the overall level of soil contamination, calculated based on CF; values > 1 indicate contamination by heavy metals. Finally, mCd was estimated as a more sensitive indicator of the overall degree of contamination, used to assess the total load of inorganic contaminants.

Igeo = Log_2_[C_s_/(1.5 × C_BL_)] (3)

EF = (C_s_/C_BL_)/(C_Fe,s_/C_Fe_,_BL_) (4)

CF = C_s_/C_BL_ (5)

PLI = (CF_s1_ × CF_s2_ × CF_s3_ × … × CF_sn_)^1/n^ (6)

mCd = ΣCF/n(7)

where C_s_ represents the measured concentration of metal *s* in the soil under evaluation; C_BL_ is the geochemical background concentration of metal *s*; C_Fe,s_ and C_Fe,BL_ are the Fe concentrations in that sample and in the geochemical background, respectively; and CF_S1_ to CF_sn_ represent the pollution factors considered; in our case, *n* = 14.

RD 9/2005 [80] on contaminated soils, together with Law 7/2022 on waste and contaminated soils for a circular economy [81], establishes that, in Spain, each Autonomous Community shall determine its own reference levels. Since the Valencian Community has not established its own reference levels, C_BL_ values were calculated as the mean of values reported for similar Mediterranean soils [11,82,83,84,85,86,87], following the uniformity criterion of “natural soils” incorporated into ISO 19258 [64]. Table 1 summarises C_BL_ and reference values (RVs).

**Table 1:** Background concentrations (C_BL_) and reference values (RVs) for elements in natural soils of the Spanish Mediterranean Region (mg kg^−1^).

	Aragón ^a^		Catalonia ^b^		Castilla-La Mancha ^c^		Murcia ^d^		MNS ^e^	MNS ^f^	Mean	SD	VC	Mean	SD	VC
Element	C_BL_	RV	C_BL_	RV	C_BL_	RV	C_BL_	RV	C_BL_	C_BL_	C_BL_			RV		
Al	4643	9827	n.a	n.a	n.a	n.a	n.a	n.a	n.a	24,469	14,556	14,019	96	9827	-	-
As	14.71	30.62	15	30	7.4	16.1	n.a	n.a	n.a	n.a	12.37	4.31	34.81	25.57	8.21	32.10
Cd	0.19	0.56	0.3	0.6	3.9 *	4.4 *	0.1	0.18	0.36	0.46	0.28	0.14	50.20	0.45	0.23	51.90
Co	8.76	18.16	15	25	5.8	20.8	6.49	13.51	8	16.60	10.11	4.56	45.11	19.37	4.81	24.86
Cr	44.34	97.67	25	85	54.8	113.4	14.11	31.21	42	45.32	37.59	15.04	40.00	81.82	35.68	43.61
Cu	15.34	34.57	20	55	10.3	27.0	15.07	25.12	12	15.1	14.64	3.33	22.75	35.42	13.68	38.61
Fe	2152	10,073	n.a	n.a	n.a	n.a	n.a	n.a	20,475	14,318	12,315	9324	76	10,073	-	-
Li	n.a	n.a	n.a	n.a	n.a	n.a	n.a	n.a	60	n.a	60	-	-	-	-	-
Mn	377	888	n.a	n.a	n.a	n.a	324.27	658.01	456	n.a	386	66	17	773	163	21
Ni	21.07	49.49	25	45	16.9	42.6	22.24	62.08	26	37.28	24.75	6.93	28.00	49.79	8.68	17.42
Pb	19.71	44.04	25	60	n.a	n.a	15.08	44.18	31	38.3	25.82	9.16	35.50	49.41	9.17	18.57
Sr	n.a	n.a	n.a	n.a	n.a	n.a	n.a	n.a	64	n.a	64	-	-	53 ^g^	-	-
V	53.61	129.25	75	135	49.9	123.2	n.a	n.a	68	n.a	61.63	11.85	19.23	129.15	5.90	4.57
Zn	43.79	189.61	60	110	35.7	86.5	57.00	120.66	56	55.91	51.40	9.50	18.48	126.69	44.31	34.97

MNS, Mediterranean natural soil; ^a^ Iribarren et al. (2008) [85]; ^b^ ARC (2009) [82]; ^c^ Jimenez-Ballesta et al. (2010) [86]; ^d^ Martínez-Martínez (2009) [87]; ^e^ Roca-Pérez et al. (2010) [11]; ^f^ Boluda (1988) [83] and Boluda et al. (1988) [84]; ^g^ Orji et al. (2025) [88]: n.a, data not available; SD, standard deviation; VC, variation coefficient; * excluded values.

### 2.4. Bioaccumulation Factor

The bioaccumulation factor (BF) was calculated to assess the capacity of plants to uptake and translocate elements [12,40,89,90,91] according to Equation (8):

BF = C_p_/C_s_ (8)

where C_p_ is the element concentration in plant tissue and C_s_ is the corresponding soil concentration.

### 2.5. Statistical Analysis

Descriptive statistics included mean, standard deviation (SD), coefficient of variation (CV), maximum (MAX), and minimum (MIN) values. Statistical analyses were performed using IBM SPSS Statistics 21 (IBM Corp., Armonk, New York, USA). Analysis of variance (ANOVA) was applied, and means were compared using Tukey’s test or Dunnett’s C test, depending on variance homogeneity. Values below LOQ were replaced with half the LOQ for statistical purposes. A Shapiro–Wilk normality test was applied to all variables. The results indicated that Al, As, Cd, Mn, Ni, Sr, V and clay exhibited a normal distribution (*p* > 0.05), whereas Co, Cr, Cu, Fe, Li, Mg, Pb, Zn, silt, sand, pH, EC, CaCO_3_, CEC, SOM and N showed non-normal distributions (*p* ≤ 0.05). Given the lack of normality in most variables, non-parametric correlations (Spearman’s rho (*p* < 0.05 *, *p* < 0.01 **)) were used to examine relationships between soil properties, among ET concentrations, between soil properties and ETs, and between soils and plants. A canonical discriminant analysis (CDA) was performed to identify variables contributing most to differences among land-use categories. Spatial representation of PLI values was generated using QGIS Desktop 3.36.1 (Zurich, Switzerland), applying IDW (Inverse Distance Weighting) interpolation. The results were visualised using an OpenStreetMap basemap data © OpenStreetMap contributors, ODbL.

## 3. Results and Discussion

### 3.1. Soil Characteristics and ET Concentrations in Soils

Soil properties were determined for each topsoil sample (Table S3), and the main descriptive statistics are summarised in Table 2. Samples S21 and S22, both Arenosols developed on aeolian–marine sands, were excluded from the statistical summary due to their markedly different texture. Overall, the soils ranged from fine textures (clay, clay loam, silty clay loam, sandy clay loam) to medium textures (loam, silt loam, sandy loam). Clay content varied between 17.8% and 47.9% (mean 31.4%); silt ranged from 20.1% to 64.9% (mean 38.2%), and sand content—excluding S21 and S22—ranged from 5.0% to 59.7% (mean 30.5%). Clay and silt showed the lowest variability (CV ≈ 26%), whereas sand exhibited the highest (CV = 46.7%). Soil pH ranged from slightly basic (7.37) to alkaline (8.65), with a mean of 8.03 and very low variability (CV = 4.5%), consistent with the Mediterranean soils developed on calcareous parent materials. Electrical conductivity (EC) showed the greatest variation (CV = 99.2%), with values between 0.13 and 2.10 dS m^−1^ (mean 0.47 dS m^−1^). Elevated EC values were recorded in the ANP rice field soils (S46–S51). Carbonate content was high across the dataset (mean 31.1%), ranging from 5.9% to 47.1% (CV = 23.5%). SOM ranged from 1.29% to 11.73% (mean 3.99%), and total N from 0.08% to 0.59% (mean 0.23%), both showing moderate to high variability. CEC values ranged from 14.5 to 26.0 cmolc kg^−1^ (mean 19.9 cmolc kg^−1^) with low variability (CV = 12.9%). These results are consistent with previous studies in Mediterranean soils [5,16,17,18,20,22,83,84,92,93,94] and indicate conditions favourable for the retention and immobilisation of trace elements.

When grouped by land use (Table S3), agricultural soils showed similar variability patterns to the overall dataset, whereas urban park (UP) and industrial park (IP) soils displayed lower variability in EC and N (CV < 6.8%), and UP soils showed relatively homogeneous carbonate content (CV = 11.6%).

The main descriptive statistics for each element in the soils are shown in Table 3. Among the ETs concentration (Table S4), Pb, Cu, Zn and Sr exhibited the highest variability (CV = 95–58%), while geochemical elements such as Al, Fe and Mn showed much lower variability (CV = 23–31%). Cd, Cr, Li and Ni displayed intermediate variability (CV = 34–38%). As and Mg showed CV values of 46.5% and 48.3%, respectively. Sandy soils (S21 and S22) consistently showed the lowest ET concentrations, reflecting their reduced adsorption capacity compared with finer-textured soils, a pattern widely documented in the literature. Overall, the results reveal substantial differences in ET concentrations across the study area, particularly for Pb, Cu, Zn and Sr. For many samples, the concentrations of these elements exceeded typical background levels for Mediterranean soils and global uncontaminated topsoils [88].

**Figure 1:**
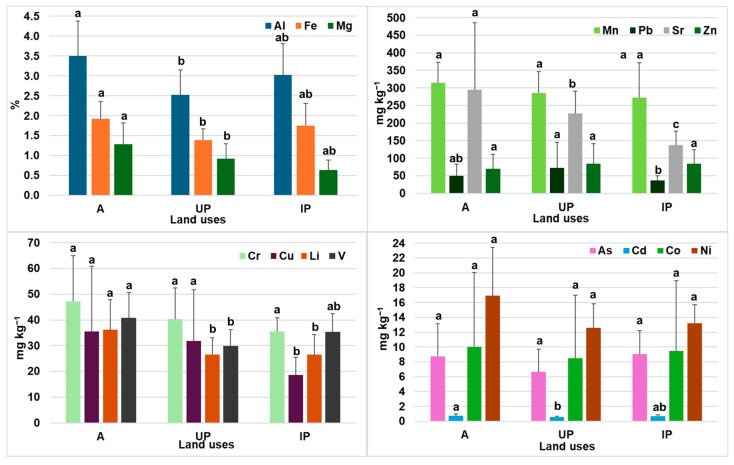
Average concentrations of elements in soils by land use and error bars (standard deviation). A: agricultural; UP: urban park; IP: industrial park. A different letter indicates statistically significant differences (*p* < 0.05) after the post hoc Dunnett’s C test.

Figure 1 shows the average concentrations of ETs in soils by land use together with the ANOVA results. The order of element abundance varied across land uses. For agricultural (A) and industrial park (IP) soils, the sequence was Al > Fe > Mg > Mn > Sr > Zn > Pb > Cr > V > Li > Cu > Ni > Co > As > Cd. In urban park (UP) soils, the sequence was Al > Fe > Mg > Mn > Sr > Zn > Pb > Cr > Cu > V > Li > Ni > As > Co > Cd, showing a change in the order for Cu, As and Ni. With the exception of As, Pb and Zn, agricultural soils exhibited higher mean concentrations than IP and UP soils. The coefficients of variation for A and UP soils followed the same pattern observed for the full dataset. The origins of Cu and Cd in soils can be linked to several anthropogenic sources, including fertilisers, pesticides and sewage sludge, with long-term fertiliser application being a well-known cause of accumulation [7,8]. Likewise, the comparatively higher As concentrations in agricultural soils may be related to the historical use of arsenic-based insecticides [51]. In contrast, UP soils showed the highest mean Pb and Zn concentrations, with the maximum values recorded at site S40 (València, Avenida Blasco Ibáñez), reaching 345.99 mg kg^−1^ for Pb and 297.66 mg kg^−1^ for Zn. These elevated levels are consistent with the site’s location in a high-traffic urban corridor. High concentrations of Zn and Pb in urban soils have been widely associated with traffic-related sources such as asphalt wear, fuel residues, brake abrasion, engine oil leakage and tyre wear [95,96]. IP soils generally showed intermediate concentrations between A and UP soils. Sample S28 (Sollana, IP), which had the highest clay content (47.9%), also exhibited the highest Fe (2.67 g 100 g^−1^) and Mn (430.01 mg kg^−1^) concentrations, consistent with previous findings linking fine-textured soils to higher metal retention [97]. ANOVA results revealed significant differences between agricultural and urban park soils for Al, Cd, Fe, Li and V, in agreement with patterns reported in earlier studies [98]. No significant differences were observed for the remaining elements.

### 3.2. ET Concentrations in Plants

The concentrations of elements in plant tissues are shown in Table S5, and Table 3 shows the main descriptive statistics. The distribution pattern differed markedly from that observed in soils. In decreasing order, mean concentrations followed the sequence Mg > Al > Fe > Sr > Mn > Zn > Cu > As > Li > Cr > Pb > Ni > V > Co > Cd. Comparing this sequence with their respective soils according to land use, changes were observed for Cr, Li, Ni and Pb in A; Cd and V in UP; and Zn, Mn, As, Cu, Cr, Li, Cd and V in IP. Aluminium ranged from 0.007% (S9, Paterna, A) to 0.309 g 100 g^−1^ (S30, Massanassa, A), while As varied between 0.37 mg kg^−1^ (S51, Sueca, A) and 20.25 mg kg^−1^ (S30, Massanassa, A). The mean concentrations of Cd, Cr and Cu were 0.17, 0.29 and 3.41 mg kg^−1^, respectively. Cu ranged from 4.38 mg kg^−1^ (S21, La Devesa, F) to 23.47 mg kg^−1^ (S31, València, A). Fe showed a mean value of 0.039 g 100 g^−1^, Li averaged 3.92 mg kg^−1^, Mg averaged 0.31 g 100 g^−1^ and Mn averaged 60.39 mg kg^−1^. Ni ranged from 0.63 mg kg^−1^ (S19, Alzira, UP) to 10.57 mg kg^−1^ (S30, Massanassa, A) and Pb from 0.30 mg kg^−1^ (S22, Sueca, A) to 17.79 mg kg^−1^ (S28, Sollana, IP). Sr and V showed mean values of 202.17 mg kg^−1^ and 1.51 mg kg^−1^, respectively. Zn ranged from 10.20 mg kg^−1^ (S50, Sueca, A) to 125.91 mg kg^−1^ (S29, Silla, A).

The concentrations of Cd, Cu, Fe, Mn, Pb and Zn fell within the ranges reported for herbaceous plants under urban anthropogenic influence, as described by Petukhov et al. [99]. Ni concentrations in rice and wheat exceeding 5 mg kg^−1^ may induce phytotoxicity [100]; sample S30 (10.57 mg kg^−1^) surpassed this threshold, likely reflecting agricultural inputs such as pesticides, fertilisers or irrigation water quality [8,16,101,102]. In this sense, Zeng et al. [102] associated plant contaminations by As, Ni, Pb, and Mn with agricultural activities, emphasising the application of agrochemicals. Additionally, previous studies indicated heavy metal contamination in soils near sample S30 [8,11,16,19], as well as soil–plant relationships in Fluvisols used for vegetable cultivation in the same area [16,17]. As observed in soils, plants growing on sandy substrates (S21, S22) generally showed the lowest ET concentrations, consistent with the reduced nutrient-holding capacity of coarse-textured soils [101].

**Figure 2:**
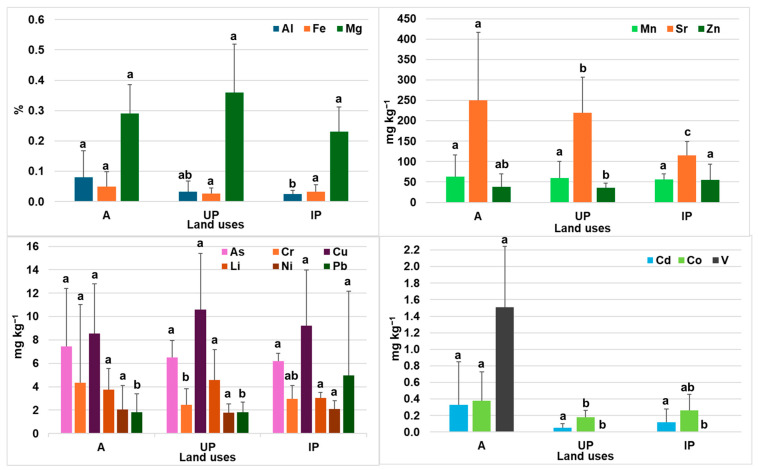
Mean concentrations of elements in plant tissues by land use (A: agricultural; UP: urban parks; IP: industrial parks). Error bars represent standard deviation. Different letters indicate statistically significant differences (*p* < 0.05) according to Tukey’s post hoc test.

Figure 2 shows ET concentrations in plants by land use. Mean concentrations of Al, As, Cd, Fe, Mn, Sr and V were higher in plants from agricultural soils than in UP and IP soils. Notably, V was detected exclusively in plants from agricultural sites, suggesting land-use-specific effects on its bioavailability. Cu was the seventh dominant element in UP and IP plants, while Co, Cr, Li, Ni, Pb and Zn showed higher concentrations in plants from industrial parks. Except for Li, agricultural soils showed the second-highest values. Statistically significant differences were found between agricultural and industrial soils for Al, Mn and Pb. Pb concentrations were markedly higher in IP plants, consistent with industrial emissions, heavy-vehicle traffic and atmospheric deposition associated with industrial activities [101,103]. Ni (2.11 mg kg^−1^) and Zn (55.47 mg kg^−1^) in IP plants exceeded those of other land uses, although values remained below those reported by Krupnova et al. [104] for industrial bioindicator species.

### 3.3. Influence of Land Use on Soil Characteristics and ET Concentrations in Soils and Plants

**Figure 3:**
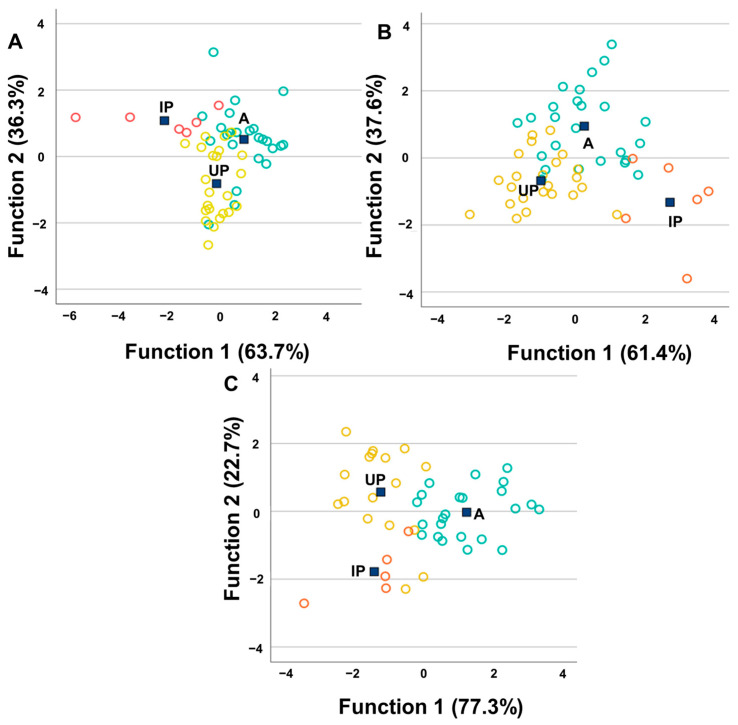
Canonical discriminant analysis of samples according to land use: (**A**) the main physical and chemical parameters in soils; (**B**) element concentrations in soils; (**C**) element concentrations in plants. The percentage of variance explained is shown in parentheses. Land uses: A, blue circles; UP, yellow circles; IP, orange circles.

Canonical discriminant analysis (CDA) was used to assess the influence of different land uses on soil properties and on the ET concentrations in soils and plants (Figure 3). Wilks’ lambda test revealed statistically significant differences between the groups (A, UP and IP) based on soil properties (λ = 0.317, *p* < 0.001; for the combination of functions 1 and 2) (Figure 3A). The two discriminant functions used explained most of the variability between the groups. The first function explained 63.7% of the total variance (with a high between-group separation of 0.708), which was associated with variables such as sand, silt and EC, whilst the second function explained 36.3% of the total variance (with a moderate to high between-group separation of 0.604), which was related to properties such as pH and carbonates. These results are consistent and indicate, as previously suggested, the differing influence of human activities on soil properties. As is well known, intensive farming and the heavy use of agrochemicals can lead, amongst other things, to the accumulation of PTEs [5,8,22,23]; urbanisation, vehicles and industrialisation can increase soil pollution and pose a risk to health.

Furthermore, the application of CDA also identified significant differences among the different land uses for ET concentrations in soils (Figure 3B) and plants (Figure 3C). In the case of ETs, function 1 explained 61.4% of the variance, with a canonical correlation of 0.759, while function 2 explained 37.6% of the variance, with a canonical correlation of 0.679. These values indicate a moderate-to-high capacity of both functions to discriminate among land uses. The Wilks’ lambda statistic indicated highly significant differences between groups when both functions were considered (λ = 0.228 and *p* < 0.001). These differences among groups have been previously reported. In their study in north-eastern China, Li et al. [105], noted that the main contamination by Cd, Cr, Cu, Hg, Ni, Pb and Zn in soils from urban areas is influenced by anthropogenic activities in cities. In addition, urban traffic constitutes an important source of PTE contamination, with Cu, Zn and Pb commonly associated with tyre and brake wear, as well as with exhaust emissions from motor vehicles [105,106,107]. On the other hand, the highest concentrations of As in soils A and IP compared to UP can be attributed to their shorter distance from the source of contamination, given that the contents of this element decrease progressively with increasing distance from that source [108]. The presence of As in A soils can be associated with commonly applied fertilisers and pesticides, while in the case of IP soils, this element is widely used as a preservative for wood and alloys, as well as being used in other types of materials [108,109]. In the case of ETs in plants, function 1 explained 77.3% of the total variance, with a canonical correlation of 0.806, whereas function 2 explained 22.7% of the variance. Wilks’ lambda identified significant differences among groups (λ = 0.230 and *p* = 0.009), while the second function was not significant (*p* = 0.394), suggesting that the differentiation of elements in plants according to land use is mainly dominated by the first canonical function. Furthermore, the centroids showed a clear separation among the different land uses. All of this indicates that land use exerts a strong influence on the multivariate accumulation pattern of elements in plants, possibly associated with differences in pollution sources, management practices, and soil conditions. Mandri et al. [110] studied the metal contents in soils and plants in areas associated with a cement plant, a landfill and a treatment facility in urban and suburban zones. In their study, they observed a clear difference in the metal accumulation profiles in plants according to land use.

### 3.4. Interactions Between Soil Characteristics and ET Concentrations in Soils

Non-parametric correlations (Spearman) revealed highly consistent patterns (highly significant correlations; ** α = 0.01 and * α = 0.05) between ETs, and among soil properties and ETs (Figure S1), showing three main trends: (i) many of the ETs were strongly inter-correlated, suggesting a shared lithogenic origin and/or similar retention processes; (ii) soil texture (clay, silt and sand) was a key determinant factor of element distributions; and (iii) SOM and chemical properties (pH, EC, CaCO_3_, CEC) modulated the mobility and retention of certain elements. These patterns are typical of natural soils and provide geochemical coherence to the dataset as a whole. Furthermore, the elements can be grouped by their levels of correlation with one another, as well as according to their ionic radii (large ions: Sr^+2^, Pb^+2^, Cd^+2^, Li^+^; medium ions: Mg^+2^, Mn^+2^, Fe^+2^, Co^+2^, Zn^+2^, Cu^+2^, Ni^+2^, V^+2^; small ions: Al^+3^, As^3+^, Cr^3+^, Fe^+3^, V^+3^), which implies analogous geochemical behaviour during paedology processes.

Regarding the ETs, a key result was the very strong positive (r ≥ 0.90 **) and very positive (0.70 ** < r < 0.90 **) correlations between Al, Fe, Li, Ni, and V, as well as between Cd, Co, Cr, Mg, and Mn, which also showed high correlations (0.40 ** < r < 0.70 **). These high correlations among lithogenic metals are widely documented in soil science [57] and are typical in soils that share a similar geological origin or retention processes. Notable specific examples were: Al-Fe: r = 0.917 **; Fe-V: r = 0.925 **; Al-V: r = 0.957 **; Al-Li: r = 0.898 **: Al-Ni: r = 0.773 **; Ni-Cr: r = 0.627 **; and Cd-Co: r = 0.613 **. This finding indicates that the elements show very marked patterns of co-occurrence, suggesting a common lithogenic origin or similar adsorption processes in the fine soil fractions. However, Cu, Pb, Sr and Zn followed a different pattern: they correlated with each other and did not correlate with Al, Fe, or V, which confirms beyond any doubt that, in many of the soils studied, their origin is not lithogenic and must be associated with exogenous sources.

With regard to the interactions between ETs and soil constituents, several correlations (*p* < 0.01) were found between the textural components (clay, silt and sand) and elements such as Al, Cd, Co, Fe, Li, Mg, Mn, Ni and V. The clay content was positively correlated with most metals (for example, Al: r = 0.798 **; Fe: r = 0.742 **; Cd: r = 0.507 **; Ni: r = 0.602 **; V: r = 0.711 **), while the sand content showed highly significant negative correlations with many metals (for example, Al: r = −0.752 **; Fe: r = −0.713 **; Li: r = −0.792 **; Mg: r = −0.674 **; Ni: r = −0.595 **; V: r = −0.702 **). Furthermore, the sand content also showed very negative correlations with clay (r = −0.767 **) and silt (r = −0.814 **). This strong relationship between these elements and soil textural components has been previously demonstrated (e.g., [5,8,11,20,22,84,111]) and reflects the influence of fine-fraction mineralogy and Fe/Al oxides. These findings indicate that soil texture is a structural factor that influences metal retention by affecting adsorption, mobility and transformation within the soil [112]. Silt, CaCO_3_ and EC were positively correlated with Li and Sr; this association between Li and Sr and the silt fraction, carbonates and soil salinity has been observed in various studies [11,88]. A significant correlation has also been found between many of the elements and the SOM content, which may be due to their high affinity and the formation of organometallic complexes, resulting in their immobility [5,8,11,12,20,22,23,84,111]. Overall, the results indicate that metal retention is strongly conditioned by soil texture and chemical properties. The clear relationships between metals and texture confirm that finer-textured soils retain more metals, whereas sandy soils exhibit lower concentrations. The observed pattern (clay/silt positive; sand negative) is fully consistent with the established edaphic literature.

Chemical properties also showed relevant relationships. Soil pH correlated negatively with SOM (r = −0.546 **), N (r = −0.757 **) and EC (r = −0.635 **), while SOM correlated positively with N (r = 0.802 **), CEC (r = 0.428 **) and EC (r = 0.559 **), consistent with the tendency of organic-rich soils to be more acidic, more saline and richer in nitrogen; in addition, CEC is strongly influenced by SOM and decreases in alkaline soils. The negative correlation between SOM and pH, and the positive correlations with N and EC, align with this behaviour. EC was associated with CaCO_3_ (r = 0.415 **) and Sr (r = 0.526 **), but negatively related to pH (r = −0.635 **). CEC correlated positively with Mg (r = 305 *) and negatively with pH (r = −0.469 **). These relationships suggest that SOM and soil chemical properties (pH, salinity, CEC) modulate metal mobility and binding forms, superimposed on a texture-driven baseline pattern. Organic matter and pH also played important roles. SOM typically enhances nutrient and metal retention but may also acidify soils; lower pH generally increases metal solubility. CEC, which is closely linked to organic matter, correlated positively with Mg and negatively with pH, consistent with changes in surface charge and functional group ionisation, indicating that CEC is strongly influenced by SOM and decreases in more alkaline soils.

Regarding the combined role of pH and SOM, SOM generally increases the retention capacity of nutrients and metals [5,8,20,22,84,113,114], although it may also contribute to soil acidification. In turn, lower pH values tend to increase the solubility of many metals [113,115]. The fact that SOM is negatively correlated with pH and positively correlated with N and EC is consistent with this interpretation. CEC showed positive correlations with Mg (r = 0.305 *) and Zn (r = 0.326 *), as well as with As, Cu, Mg, Sr and Zn. This relationship has been reported in previous studies; Li et al. [112] noted that this parameter promotes the retention of these elements, whereas its relationship with pH was negative.

**Figure 4:**
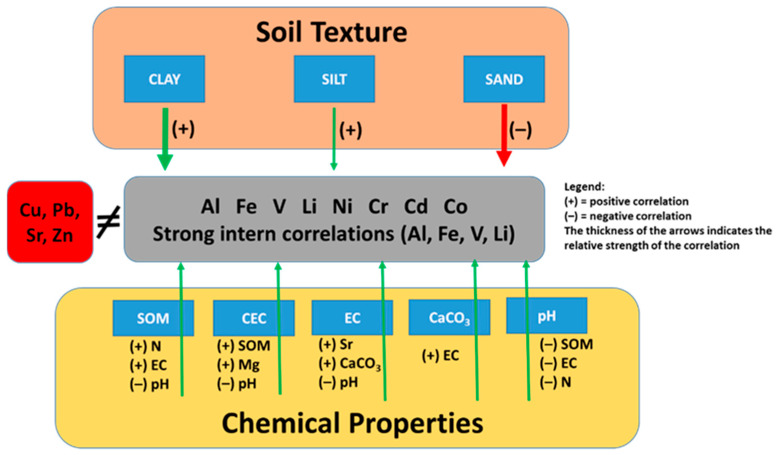
Conceptual diagram illustrating the main relationships between soil constituents, metal concentrations and chemical properties.

Taken together, the results are statistically robust and align well with findings reported for Mediterranean and similar soils, where texture exerts strong control, lithogenic metals co-occur, and pH, SOM and salinity modulate mobility. Figure 4 shows a conceptual diagram that illustrates the main relationships between soil constituents, metal concentrations and chemical properties. It can be observed that the fine fraction (clay and silt) shows positive correlations with most metals (Al, Fe, V, Li, Ni, Cr, Cd, Co), whereas sand shows negative correlations. Metals form a highly inter-correlated block, particularly Al–Fe–V–Li. In contrast, Cu, Pb, Sr and Zn show completely different behaviour. Chemical properties modulate mobility and retention: SOM increases N and EC and reduces pH; EC is associated with Sr and CaCO_3_ and decreases with pH and N; CEC increases with SOM and Mg and decreases with pH.

### 3.5. Correlation Among Element Contents in Plants and the Soil–Plant System

Figure S2 shows the correlations obtained between the concentration of elements in the plant material and the correlation between the soil–plant system. The correlations showed well-defined patterns. One group, consisting of Al, Fe, Co, Cr, Ni, and Mn, showed high and significant correlations, suggesting related absorption or translocation mechanisms in the plant. A second group, consisting of Cd, Zn, Pb, and Cu, also showed associations. More specifically, high correlations were observed between Al and Fe, with r = 0.720 **; this could be explained by the fact that both come from the same mineral fraction of the soil, which is susceptible to being released and thus absorbed by plants in a similar way [116,117]. Likewise, the positive correlation between Pb and Ni (r = 0.546 **), as well as between Pb and Zn (r = 0.645 **), could be associated with the fact that the accumulation of Pb in plants does not occur in isolation and could be conditioned by common environmental circumstances [110]. By correlating the concentrations of elements in soils with their respective concentrations in plants, varied patterns were observed; for example, As (r = 0.465 **), Cd (r = 0.489 **) and Cu (r = 0.310 *) showed positive correlations, indicating that higher total concentrations in soil may be associated with higher concentrations in plant tissues [118]. As, in turn, strongly depends on its chemical form in soil, as well as on pH and organic matter content; therefore, positive correlations between As in soil and in plants usually indicate that the plant is absorbing the bioavailable fraction of As present in the soil [119]. In contrast, elements such as Fe, Mg, Mn, Ni, Pb and Zn did not show significant correlations, which may suggest that their transfer is mediated by the bioavailability of these elements in the soil.

Furthermore, correlations were found between Mg_s_ and Cd_p_ (r = 0.631 **), Pb_s_ and Cu_p_ (r = 0.631 **) or Fe_s_ and Cd_p_ (r = 0.366 *). These correlations can be explained by the non-specific nature of root transport systems. Plants rely on transporters to absorb essential nutrients such as Ca, Fe, Mn and Zn; however, under conditions of high soil concentrations, these systems may facilitate the uptake of other elements. This phenomenon is relevant for metals such as Cd and metalloids such as As, which have no known biological function. In addition, it has been demonstrated that Cd uptake occurs primarily via transporters intended for essential divalent cations, including Ca, Fe, Mn and Zn. In this context, the iron transporter IRT1 has been identified as a significant contributor to Cd entry into roots, which may generate a positive correlation between the availability of Fe or Zn in the soil and Cd accumulation in plants [120].

Taken together, the contrasting patterns observed between soils and plants highlight the combined influence of land use, soil texture and chemical properties on ET concentrations. These differences justify a deeper examination of contamination indices and soil–plant transfer mechanisms, which are addressed in the following sections.

### 3.6. Assessing Soil Contamination

**Figure 5:**
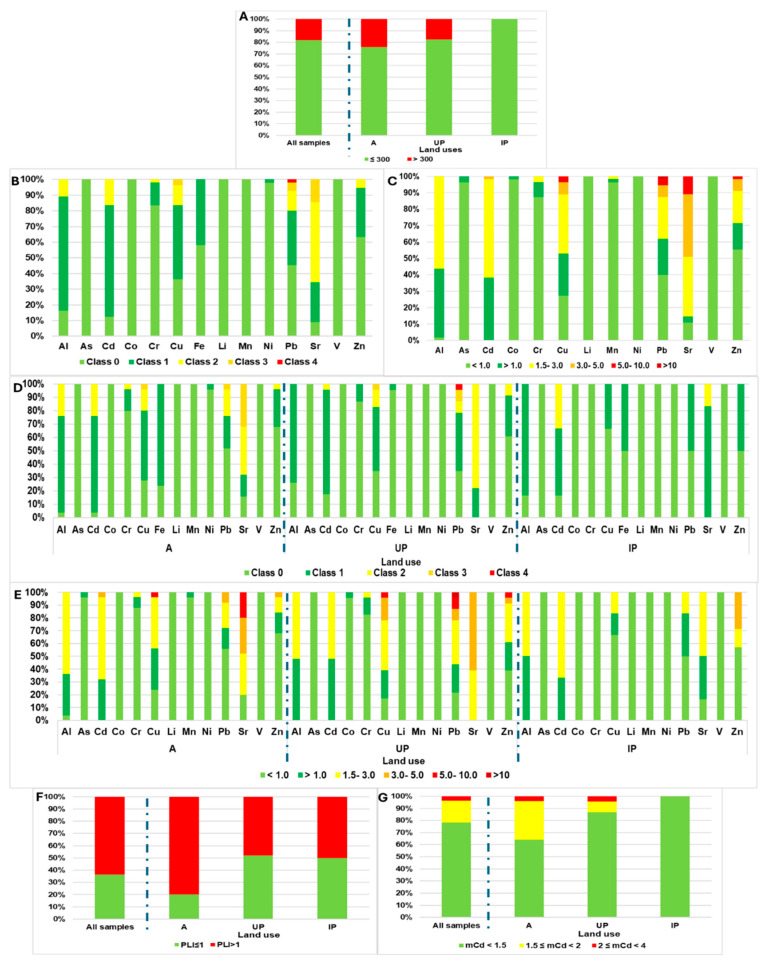
Results after interpreting soil contamination indices by land use. (**A**) Zinc equivalent (ZnEq); (**B**) geoacumulation index (Igeo); (**C**) enrichment factor (EF); (**D**) Igeo by land uses; (**E**) EF by land uses; (**F**) pollution load index (PLI); (**G**) modified degree of contamination (mC_d_).

The application of soil contamination indices is fundamental for transforming complex chemical data into actionable information for environmental decision-making. These indices allow us to differentiate between naturally occurring (lithogenic) concentrations and those resulting from human activities (anthropogenic). Figure 5 shows the results after applying contamination indices to assess soil quality using the background levels obtained in this study (Table 1) and the interpretation shown in Table S2. Figure 5A shows the ZnEq values for the entire set of samples and by land use. Of the total samples, 18.8% had values exceeding 300 mg kg^−1^, which would suggest that these soils could be considered potentially toxic [18]. For agricultural soils, 24% of the samples exceeded this threshold value, followed by UP soils (17.4%), whilst no IP soils were found to be toxic. The results obtained after Igeo application are shown in Figure 5B,D. In general, the Igeo values ranged from Class 0 (virtually uncontaminated) for As, Co, Li, Mn and V to Classes 3 and 4 (moderately to highly contaminated), and highly contaminated (Class 4) for Cu, Pb, Sr and Zn (Figure 5B). The A and UP soils exhibited the highest values for Cu, Pb, Sr and Zn (Figure 5D). The presence of elevated Cu and Pb values observed in UP soils is consistent with results from other urban environments [43,95]. In addition, in a comparative study of urban green spaces and agricultural soils in Iran, Mirzaei et al. [98] observed that a higher percentage of agricultural soil samples were classified in higher classes compared with urban soils. Pb was the exception, for which 3.7% of the urban samples were classified in Class 3, compared with 2.12% of the samples from agricultural soils. On the other hand, the degrees of contamination associated with industrial activities and other anthropogenic sources were similar to those reported in Gijón (Spain), which presented values within comparable ranges for Al, As, Fe, V and Zn [26]. The EF index results for all soil samples, broken down by land use, are shown in Figure 5C. These results demonstrate the effect of land use on the degree of enrichment in Pb, Cu, Sr, and Zn, identified as the main indicators of anthropogenic pressure. Furthermore, Li, Ni and V were classified in the category of possible metal mobilisation or depletion. In contrast, Cu, Pb, Sr and Zn accounted for approximately 10% of the samples classified within the category of severe anthropogenic origin, with Pb even reaching this category. Likewise, land use A, with respect to Cu, Pb and Sr, showed a higher proportion of samples in intermediate and high ranges, which suggests a moderate to severe anthropogenic influence (Figure 5E). With regard to UP soils, Pb again stood out. These results were slightly higher than those reported by Tamim et al. [60] and Tomczyk et al. [13] in agricultural and urban soils for elements such as Cu, Pb and Zn. However, the values for Cd, V and Sr showed similar results. To assess the overall level of contamination by PTEs in the study area and thereby predict the environmental status of soils under different land-use conditions, the PLI (Figure 5F) and mCd (Figure 5G) indices were applied. Overall, 65% of the samples analysed had PLI values ≥ 1, indicating widespread contamination and, consequently, the progressive deterioration of soil quality [60,61]. Likewise, 80% of the A soils presented PLI values ≥ 1, followed by IP soils, with 50% of the samples; UP soils were in third place, with 48% of the analysed samples. Regarding the mCd index, according to the criterion proposed by Vineethkumar et al. [61], 22% of the soils would be considered moderately to slightly contaminated, and the contamination dynamics according to land use were as follows: A, 36% of the samples; UP, 13% of the samples; and IP, no contamination was observed. In a study of 51 agricultural soils in India, Mandal et al. [121] obtained a median value of 1.51 for the modified contamination index, indicating that contamination by the studied elements was low in the sampling area, as also observed in this study.

**Figure 6:**
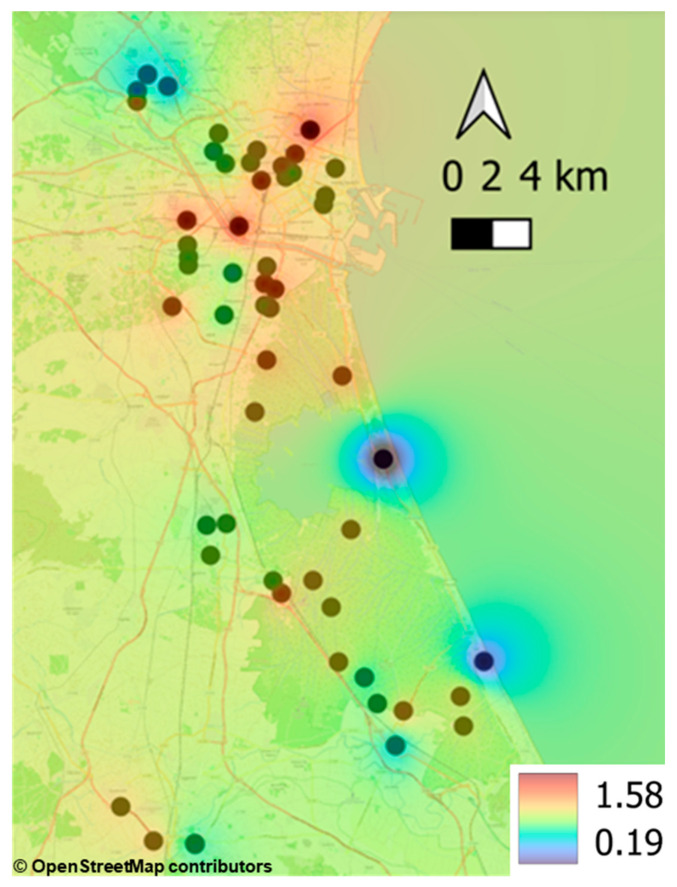
Spatial distribution of the PLI index of the soil samples. Base map © OpenStreetMap contributors, ODbL. Available online: https://www.openstreetmap.org/#map=11/39.4118/-0.3365 (accessed on 14 April 2026).

Moreover, an IDW analysis was carried out using the PLI results in order to examine the spatial distribution of ET concentrations without the need for prior geostatistical modelling (Figure 6). A trend can be observed in the distribution of the PLI values, which are higher in the most densely populated areas, such as Alboraia and the city of València, as well as Benetússer, Alfafar, Sedaví, Catarroja and Massanassa, which were recently affected by the DANA event of 29 October 2024. This pattern is consistent with the results reported by other authors [122,123,124].

On the other hand, comparing our results with the C_BL_ and RV proposed in Table 1, we observe that, for Cu, 85% (47 samples) of the soils exceeded the background level, and 20% (11 samples) exceeded the reference value. In the case of Pb, 41% exceeded the C_BL_, and 38% exceeded the RV. Regarding Sr, all soils exceeded the C_BL_ established by Roca-Perez et al. [11] and the threshold of 53 mg kg^−1^ indicated by Orji et al. [88]. Our results show that some soils exceed the background levels indicated in Table 1, as well as the reference value of 298 mg kg^−1^ reported by Roca-Pérez et al. [11]. Moreover, the origin of these elevated concentrations is most likely attributable to exogenous sources, primarily the use of agrochemicals [11,57,58]. In contrast, for soils with concentrations within normal levels, their origin is likely associated with the calcareous nature of the parent material and the dynamics of the calcium ion (Figure 4 and Figure S1). All this suggests that further research on the RV for this element in our soils is needed. In the case of Zn, 55% of the soils showed levels above the BL, and 9% (five samples) were above the RV. Likewise, when comparing our results with the generic reference levels indicated in the legislation of Autonomous Communities such as Aragón, Cataluña and Madrid, the concentrations measured in the soils for Cd, Cr, Cu, Pb and Zn exceeded the generic reference values at some of the sampling sites [82,85,125]. In accordance with these regulations, agricultural soils are classified under the ‘other’ category, with particular concern regarding contamination by Cu and Pb. About Pb, soils A, with an average value of 49.49 mg kg^−1^, exceeded the limit proposed by Aragon of 45 mg kg^−1^ [85]. Similarly, this occurred with Cu, whose average concentration (35.50 mg kg^−1^) exceeded the limit value of 28 mg kg^−1^ [85]. Regarding Zn, sample S44, from Alboraia, presented a concentration of 226.50 mg kg^−1^, exceeding the limit established by Catalonia legislation [82]. Cr in sample S45 (València) recorded a concentration of 110.26 mg kg^−1^, exceeding the reference value of 90 mg kg^−1^ proposed by the legislation of the Madrid Community [125]. Regarding urban soils, samples S6 (Paterna), S32 (Valencia), S33 (Valencia), S35 (Valencia), S40 (Valencia), S42 (Valencia), and S53 (Sollana) exceeded the Pb limit proposed by Catalonia of 60 mg kg^−1^, and sample S6 (345.99 mg kg^−1^) exceeded the limit set by other Autonomous Communities (270 mg kg^−1^ defined by Aragón and Madrid Communities). Surprisingly, it is worth noting that all samples taken from industrial sites show concentrations below these standards, as the limits set for industrial sites are much more lenient.

### 3.7. Soil Plant Transfer

**Figure 7:**
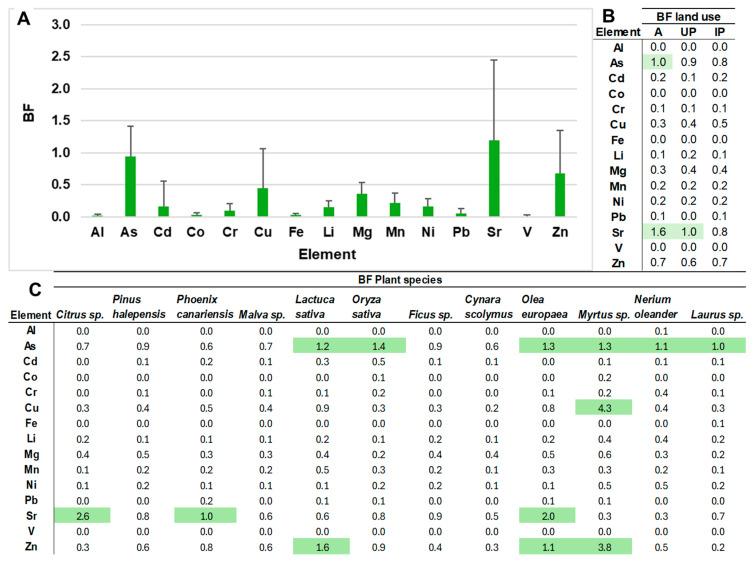
Bioaccumulation factor (BF): (**A**) mean for all samples (error bars indicate standard deviation); (**B**) according to land use; (**C**) according to plant species. Values highlighted in green indicate BF ≥ 1.

The transfer factor is a critical index that measures the efficiency with which a plant absorbs a specific element (such as Al, As, Cd, Cu, Fe, Li, Ni or V) from the soil into its tissues. Its application is fundamentally important for food safety assessment, phytoremediation potential, bioavailability versus total concentration, and environmental risk management [12,40,89,90,91,126]. In this study, the bioaccumulation factor (BF) was calculated (Figure 7) to assess the capacity of plants to uptake and translocate ETs [40,89,90,91]. Figure 7A shows the mean BF values for all analysed samples. Overall, most elements exhibited very low bioaccumulation rates (BF < 1), indicating a limited capacity for transfer from soil to plant. Exceptions were As, Cu, Sr and Zn, whose values showed that some plants are capable of bioaccumulating these elements. These results are consistent with those reported by Eben et al. [126], who identified the ability of these elements to accumulate in the aerial parts of herbaceous species, with BF values ≥ 1. In the case of Sr, soils in arid and semi-arid regions contain high concentrations of calcium carbonate, which favours Sr retention due to its geochemical similarity to Ca; both belong to Group II of the alkaline earth metals and have similar ionic charges and radii. Furthermore, Sr tends to accumulate preferentially in surface horizons. All of this results in greater Sr migration, which depends on soil factors as well as the plant’s ability to incorporate this element into metabolic processes analogous to those involving Ca (e.g., maintenance of enzymatic activity) [11,127,128]. Regarding As, its presence in plants is expected, since under aerobic conditions, As occurs in soil mainly as arsenate, a more bioavailable species. Arsenate is absorbed by plants through phosphate transport systems due to its structural similarity to this anion, generating direct competition during root uptake [129]. Conversely, elements such as Pb showed low bioavailability in all cases. This can be attributed to the strong adsorption of Pb by soil constituents, which limits its mobility, uptake and bioavailability to plants [91,130,131].

With respect to soil use, the BF values are shown in Figure 7B. As exhibited BF = 1 in agricultural soils, whereas Sr showed values of 1.6 and 1.0 in A and UP soils, respectively. Low BF values were previously reported by Xu et al. [131] in their study on heavy metal accumulation in crop grains under different agricultural land-use patterns; they observed weak correlations between total metal concentrations in soil and their content in grain. These results suggest the existence of biological regulatory mechanisms that limit metal transfer to the aerial parts of plants.

Figure 7C shows the BF values as a function of plant species. *Myrtus* sp. clearly stood out from the other species, exhibiting a notable number of bioaccumulated elements (As, Cu and Zn). This was due to sample S21, which showed very high BF values, likely explained by its sandy texture and low CEC. *Olea europaea* showed BF > 1 for As, Sr and Zn. *Lactuca sativa*, *Oryza sativa*, *Nerium oleander* and *Laurus* sp. also exhibited elevated BF values for As. *Citrus* sp. showed bioaccumulation of Sr. The uptake and accumulation of inorganic pollutants in plants are influenced by both soil properties and plant physiological characteristics and species [132]. Regarding plant type, Aina et al. [133] evaluated the BF of heavy metals in *Lactuca sativa* and observed high Zn accumulation, consistent with the results obtained here. Likewise, the authors suggested that broad-leaf vegetables show a high affinity for metal accumulation due to their high transpiration rate and large leaf surface area during growth.

## 4. Conclusions

This study provides a comprehensive assessment of major and potentially toxic elements in soils and plants across agricultural, urban and industrial areas of the València region. Soil properties showed substantial spatial heterogeneity, strongly influenced by land use, particularly in relation to texture, organic matter, salinity and nitrogen. Element concentrations exhibited marked variability, with Cu, Pb, Sr and Zn showing the highest dispersion and clear evidence of anthropogenic enrichment, especially in agricultural and urban environments, confirmed by canonical discriminant analysis. In contrast, lithogenic elements displayed more stable patterns consistent with Mediterranean soils developed on calcareous parent materials. Non-parametric correlations between elements, texture and soil chemical properties reveal a pattern highly consistent with the established soil science literature. Lithogenic metals (Al, Fe, V, Li, Ni, Cr, Cd, Co) show strong co-occurrence, suggesting a shared origin and preferential retention in the fine fraction. Texture emerges as the primary structural factor: clay- and silt-rich soils retain higher metal concentrations, whereas sandy soils show lower levels. Chemical properties—particularly pH, SOM, EC and CEC—modulate metal mobility and availability, reinforcing the integrated role of texture, organic matter and soil chemistry in controlling metal distribution. Overall, the results indicate that soil quality and geochemical behaviour are strongly governed by the interaction between texture, metals and chemical properties. Contamination indices (ZnEq, Igeo, EF, PLI and mCd) indicated moderate to severe contamination risks, with several elements (Cd, Cr, Cu, Pb, Sr, Zn) exceeding regional reference values. Spatial analysis revealed higher contamination gradients in densely populated areas. Plant tissues presented high levels of Mg, Al, Fe and Mn, while isolated elevated concentrations of Ni and Pb in species such as myrtle, mallow, laurel and rice pointed to localised hotspots of bioavailability. Bioaccumulation indices indicated notable plant uptake of As, Cu, Sr and Zn, highlighting their potential for trophic transfer. Land use significantly influenced As and Sr accumulation in plants, suggesting differentiated exposure pathways across the landscape. Our results have important implications for soil management in the area: (i) priority should be given to monitoring Cd, Cr, Cu, Pb, Sr and Zn due to their consistent enrichment and regulatory exceedances; (ii) agricultural and urban soils require targeted risk-based management to minimise human and ecological exposure; (iii) contamination maps should guide land-use planning, especially in densely populated areas; (iv) elevated bioaccumulation of certain elements in some plant species highlight the need to assess food chain transfer; and (v) bioindicator species may support early detection of hotspots. Future research should evaluate post-flood contaminant redistribution and establish long-term soil–plant monitoring frameworks.

## Figures and Tables

**Table 2:** Main descriptive statistics for soil properties.

Parameter	Mean	SD	Min	Max	CV
Clay (%)	31.4	8.2	17.8	47.9	26.2
Silt (%)	38.2	10.0	20.1	64.9	26.3
Sand (%)	30.5	14.2	5.0	59.7	46.7
pH (H_2_O)	8.03	0.36	7.37	8.65	4.5
EC (dS m^−1^ 25 °C)	0.47	0.46	0.13	2.10	99.2
CaCO_3_ (%)	31.1	7.3	5.9	47.1	23.5
CEC (cmol_c_ kg^−1^)	19.9	2.6	14.5	26.0	12.9
SOM (%)	3.99	1.7	1.29	11.73	43.7
N (%)	0.23	0.11	0.08	0.59	47.4

EC, electrical conductivity; CEC, cation exchange capacity; SOM, soil organic matter; N, total nitrogen; SD, standard deviation; Min, minimum value; Max, maximum value; CV, coefficient of variation. n = 53.

**Table 3:** Main descriptive statistics for ET concentrations in soils and plants (mg kg^−1^).

	Soils n = 55					Plants n = 47				
Element	Mean	SD	Min	Max	CV	Mean	SD	Min	Max	CV
Al ^a^	2.99	0.95	0.34	4.45	31.72	0.054	0.069	0.007	0.309	127.78
As	7.80	3.63	<LOD	14.22	46.52	6.86	3.63	0.37	20.25	52.84
Cd	0.63	0.22	0.12	1.35	34.53	0.17	0.34	<LOD	1.43	204.90
Co	9.05	2.49	<LOD	14.60	27.56	0.29	0.28	<LOD	1.30	95.95
Cr	42.25	15.74	4.28	110.26	37.25	3.41	4.85	<LOD	33.38	142.36
Cu	31.49	22.30	1.02	141.73	70.82	9.31	4.54	4.38	23.47	48.81
Fe ^a^	1.65	0.49	0.24	2.67	29.99	0.039	0.038	0.006	0.196	98.51
Li	30.60	10.88	4.04	57.50	35.56	3.92	2.10	<LOD	9.63	53.50
Mg ^a^	1.04	0.50	0.29	2.22	48.33	0.31	0.13	0.13	0.78	41.62
Mn	294.86	68.48	122.23	430.01	23.22	60.39	44.85	22.45	234.10	74.27
Ni	14.42	5.58	1.55	37.50	38.70	1.92	1.51	0.63	10.57	78.66
Pb	56.91	54.06	9.17	345.99	94.99	2.14	2.63	0.30	17.79	122.48
Sr	245.89	144.52	66.19	696.66	58.77	202.17	136.72	20.87	509.70	67.63
V	35.08	10.34	5.80	55.49	29.48	1.51	0.73	<LOD	2.89	48.80
Zn	76.08	48.91	7.81	297.66	64.28	38.86	26.18	10.20	125.91	67.37

Elements in mg kg^−1^ except elements ^a^ in g/100 g; SD, standard deviation; Min: minimum value; Max: maximum value; CV, coefficient of variation; LOD, limit of detection (LOD < 0.01 mg kg^−1^).

## Data Availability

The original contributions presented in this study are included in this article/Supplementary Materials. Further inquiries can be directed to the corresponding author.

## Supplementary Materials

The following supporting information can be downloaded at https://www.mdpi.com/article/10.3390/toxics14050353/s1: Figure S1: Spearman correlation matrix for soil characteristics and ETs (EC, electrical conductivity; CEC, cation exchange capacity; SOM, soil organic matter; N, total nitrogen); ** α = 0.01 and * α = 0.05; Figure S2: Spearman correlation coefficients among element concentrations in plants and between soil–plant system; Table S1: Sample location and soil–plant identification; Table S2: Interpretation of contamination indices; Table S3: Soil characteristics; Table S4: Soil element concentrations; Table S5: Plant element concentrations.
