# Supplementary material for: Soil–Plant Transfer and Environmental Levels of Potentially Toxic Elements in Agricultural, Urban and Industrial Areas of the València Region (Eastern Spain)
Source: Toxics. 2026 Apr 22;14(5):353. doi: 10.3390/toxics14050353 (PMC13211046; doi:10.3390/toxics14050353)
Supplement: Supplementary file 1 [file toxics-14-00353-s001.zip › toxics-4265942-supplementary.pdf]

Supplementary Materials

	Al	As	Cd	Co	Cr	Cu	Fe	Li	Mg	Mn	Ni	Pb	Sr	V	Zn	Clay	Silt	Sand	pH	EC	$CaCO_3$	CEC	SOM	N
As	,340*	--																						
Cd	,660**	,539**	--																					
Co	,593**	,774**	,613**	--																				
Cr	,410**	0,191	,540**	,338*	--																			
Cu	0,113	,326*	,532**	0,144	,617**	--																		
Fe	,937**	,530**	,741**	,743**	,407**	0,186	--																	
Li	,898**	0,196	,581**	,369**	,461**	0,246	,803**	--																
Mg	,516**	0,168	,346**	0,202	0,257	,296*	,527**	,662**	--															
Mn	,401**	,581**	,497**	,701**	,279*	,296*	,562**	,387**	,494**	--														
Ni	,773**	0,172	,641**	,566**	,627**	0,197	,727**	,763**	,374**	,377**	--													
Pb	-0,170	0,071	0,207	0,110	,423**	,491**	-0,106	-0,066	-0,119	0,228	0,182	--												
Sr	0,205	-,292*	0,179	0,027	,488**	,376**	0,092	,455**	,544**	0,189	,431**	,311*	--											
V	,957**	,333*	,640**	,689**	,402**	0,073	,925**	,806**	,515**	,425**	,791**	-0,144	0,234	--										
Zn	-0,228	0,220	,294*	0,133	,463**	,639**	-0,149	-0,143	-0,172	0,179	0,069	,667**	0,198	-0,244	--									
Clay	,798**	,346*	,507**	,437**	0,103	0,012	,742**	,800**	,489**	,424**	,602**	-0,210	0,120	,711**	-,270*	--								
Silt	,485**	0,057	,461**	0,251	,324*	,357**	,485**	,546**	,697**	,319*	,392**	-0,012	,401**	,479**	-0,175	,317*	--							
Sand	-,752**	-0,174	-,557**	-,336*	-0,200	-0,185	-,709**	-,792**	-,672**	-,369**	-,595**	0,131	-,285*	-,702**	,322*	-,767**	-,814**	--						
pH	0,221	,393**	0,178	,432**	-0,163	-,369**	0,232	0,033	-0,209	0,161	0,192	-0,242	-,413**	0,223	-,300*	,290*	-0,067	-0,160	--					
EC	0,174	-,425**	0,015	-,450**	0,240	0,257	0,012	,353**	,323*	-,356**	0,101	-0,023	,526**	0,092	-0,041	0,075	,339*	-,266*	-,635**	--				
$CaCO_3$	0,234	-0,185	0,087	-0,196	0,114	0,238	0,145	,378**	,547**	0,097	0,122	0,012	,437**	0,240	-0,126	,274*	,525**	-,516**	-0,250	,415**	--			
CEC	-0,003	0,076	0,124	0,089	,288*	,328*	0,046	0,058	,305*	0,205	0,006	0,151	,323*	0,006	,326*	-0,042	0,198	-0,027	-,469**	,326*	0,106	--		
SOM	-0,084	-,329*	-0,041	-,381**	0,156	0,118	-0,132	-0,048	0,057	-,393**	-0,169	-0,030	0,233	-0,096	0,089	-,272*	0,212	0,029	-,546**	,559**	0,088	,428**	--	
N	-0,071	-,416**	-0,034	-,426**	,290*	,281*	-0,165	0,095	0,124	-,348**	-0,061	0,129	,505**	-0,109	0,227	-0,247	0,092	0,112	-,757**	,733**	0,116	,518**	,802**	--

** (α= 0.01) and * (α= 0.05); n = 55

LEGEND
- Strong positive
- Very positive
- Positive
- Very negative
- Negative
- No correlation

Figure S1. Spearman correlation matrix for soil characteristics and ETs (EC, electrical conductivity; CEC, cation exchange capacity; SOM, soil organic matter; N, total nitrogen; **(α = 0.01) and *(α = 0.05).

	Al_p	As_p	Cd_p	Co_p	Cr_p	Cu_p	Fe_p	Li_p	Mg_p	Mn_p	Ni_p	Pb_p	Sr_p	V_p	Zn_p
Al_p	1														
As_p	-0.009	1													
Cd_p	0.518**	0.414*	1												
Co_p	0.823**	0.066	0.588**	1											
Cr_p	0.472**	0.175	0.385*	0.599**	1										
Cu_p	0.232	0.230	0.456**	0.299*	0.025	1									
Fe_p	0.720**	0.139	0.576**	0.866*	0.734**	0.323*	1								
Li_p	-0.112	0.625*	0.411*	-0.123	-0.059	0.297*	-0.11	1							
Mg_p	-0.075	0.547**	0.298	-0.061	-0.085	0.26	-0.004	0.343*	1						
Mn_p	0.283	-0.007	0.422*	0.590**	0.519**	0.168	0.654**	-0.156	0.071	1					
Ni_p	0.391**	0.322*	0.446*	0.602**	0.629**	0.221	0.587**	0.06	0.153	0.415**	1				
Pb_p	0.257	0.545**	0.522**	0.459**	0.415**	0.445**	0.602**	0.365*	0.259	0.423**	0.546**	1			
Sr_p	-0.260	0.684**	0.000	-0.278	-0.242	0.104	-0.323*	0.581*	0.458**	-0.427**	0.026	0.12	1		
V_p	0.833**	0.762*	-1.000	0.898**	0.524	0.905**	0.833*	0.762*	0.619	0.500	0.548	0.762*	0.524	1	
Zn_p	0.227	0.444**	0.688**	0.352*	0.303*	0.535**	0.424**	0.272	0.161	0.369*	0.363*	0.645**	-0.073	0.571	1
Al_s	0.159	0.096	0.397*	0.303*	0.212	-0.101	0.321*	-0.07	0.11	0.261	0.05	-0.011	0.105	-0.333	-0.183
As_s	-0.032	0.465**	0.351*	-0.087	-0.082	0.137	-0.158	0.400**	0.252	-0.272	0.013	0.077	0.548**	0.179	0.132
Cd_s	0.261	0.274	0.489**	0.306*	0.225	0.147	0.274	0.19	0.126	0.114	0.185	0.142	0.268	0.595	0.085
Co_s	-0.200	0.325*	0.057	-0.209	-0.139	0.037	-0.177	0.293	0.319*	-0.072	-0.163	-0.049	0.439**	0.886*	-0.126
Cr_s	0.334*	0.023	0.216	0.337*	0.121	0.025	0.122	0.203	0.042	0.118	0.138	-0.043	0.189	0.024	-0.198
Cu_s	0.328*	0.154	0.443*	0.332*	-0.024	0.310*	0.14	0.231	0.191	-0.096	0.26	0.12	0.336*	-0.238	0.031
Fe_s	0.175	0.139	0.366*	0.263	0.133	-0.036	0.253	-0.018	0.099	0.137	0.013	-0.005	0.170	-0.262	-0.155
Li_s	0.294*	-0.036	0.443*	0.455**	0.242	0.031	0.405**	-0.093	0.065	0.330*	0.126	0.010	0.026	-0.571	-0.153
Mg_s	0.328*	0.044	0.631**	0.442**	0.142	0.302*	0.350*	0.025	0.278	0.281	0.16	0.046	0.066	-0.381	-0.037
Mn_s	0.036	0.113	0.294	0.033	-0.141	0.308*	-0.007	0.201	0.156	-0.061	-0.134	0.083	0.257	0.095	-0.07
Ni_s	0.156	-0.057	0.278	0.275	0.155	-0.035	0.187	-0.048	0.012	0.271	0.036	-0.113	0.010	0.024	-0.214
Pb_s	0.112	-0.036	-0.051	0.014	-0.210	0.359*	-0.045	0.153	-0.038	-0.096	-0.041	0.157	0.139	0.69	-0.017
Sr_s	0.356*	-0.271	0.301	0.397**	0.199	0.158	0.350*	-0.079	0.124	0.367*	0.229	0.033	-0.120	-0.524	-0.218
V_s	0.099	0.068	0.373*	0.265	0.179	-0.14	0.267	-0.113	0.126	0.27	0.024	-0.04	0.097	-0.19	-0.216
Zn_s	0.104	0.123	0.142	-0.033	-0.181	0.340*	-0.114	0.342*	0.102	-0.183	0.15	0.095	0.263	0.143	0.122

p, plant; s, soil. ** (α= 0.01) and * (α= 0.05)

LEGEND
- Strong positive
- Very positive
- Positive
- Negative
- No correlation

Figure S2. Spearman correlations coefficients among element concentrations in plants, and between soil-plant system.

Table S1. Sample location and soil-plant identification.

Samples	Coordinates (UTM)	Municipality	Land use	Parent material/Possible Reference soil group (WRB, 2022)	Plant sample
S1	30 S 0721367 - 4369740	València	A	Alluvial silt deposits/Fluvisol	*Citrus sp.*
S2	30 S 0720611 - 4365311	Catarroja	A	Colluvial/Calcisol	*Citrus sp.*
S3	30 S 0721394 - 4368472	Picanya	IP	Alluvial silt deposits/Fluvisol	*Pinus halepensis*
S4	30 S 0721448 - 4367850	Picanya	UP	Alluvial silt deposits/Fluvisol	*Pinus halepensis*
S5	30 S 0721437- 4367446	Picanya	A	Colluvial/Calcisol	*Citrus sp.*
S6	30 S 0720384 - 4376640	Paterna	UP	Limestone/Calcisol	*Pinus halepensis*
S7	30 S 0719336 - 4377243	Paterna	IP	Limestone/Calcisol	*Pinus halepensis*
S8	30 S 0718815 - 4376405	Paterna	UP	Colluvial/Anthrosol	*Olea europaea*
S9	30 S 0718798 - 4375867	Paterna	A	Alluvial silt deposits/Fluvisol	*Citrus sp.*
S10	30 S 0725479 - 4367364	Sedaví	UP	Alluvial silt deposits/Fluvisol	*Phoenix canariensis*
S11	30 S 0725359 - 4366488	Alfafar	UP	Alluvial silt deposits/Fluvisol-Anthrosol	*Malva sp.*
S12	30 S 0725373 - 4365375	Massanassa	IP	Alluvial silt deposits/Anthrosol	*Pinus halepensis*
S13	30 S 0725895 - 4366202	Massanassa	A	Alluvial silt deposits/Anthrosol	*Cynara scolymus*
S14	30 S 0723399 - 4354140	Almussafes	IP	Limestone/Calcisol	*Pinus halepensis*
S15	30 S 0722385 - 4354056	Almussafes	IP	Alluvial silt deposits/Anthrosol	*na*
S16	30 S 0722580 - 4352508	Almussafes	A	Limestone/Calcisol	*Citrus sp.*
S17	30 S 0717962 - 4339579	Guadassuar	A	Black Albufera silts/Anthrosol	*Citrus sp.*
S18	30 S 0719649 - 4337809	Alzira	A	Alluvial silt deposits/Fluvisol	*Citrus sp.*
S19	30 S 0721784 - 4337637	Alzira	UP	Alluvial silt deposits/Fluvisol	*Lactuca sativa*
S20	30 S 0721772 - 4337680	Alzira	UP	Alluvial silt deposits/Fluvisol	*Lactuca sativa*
S21	30 S 0731469 - 4357461	La Devesa	F	Aeolian-marine sands/Arenosol	*Myrtus communis*
S22	30 S 0736650 - 4347052	Sueca	A	Aeolian-marine sands/Arenosol	*Nerium oleander*
S23	30 S 0735441 - 4345252	Sueca	A	Black Albufera silts	*Oryza sativa*
S24	30 S 0732503 - 4344510	Sueca	A	Alluvial silt deposits/Fluvisol	na
S25	30 S 0731167 - 4344882	Sueca	A	Black Albufera silts/Anthrosol	*Citrus sp.*
S26	30 S 0729169 - 4347023	Sueca	A	Black Albufera silts/Fluvisol-Anthrosol	*Oryza sativa*
S27	30 S 0728790 - 4349841	Sueca	A	Black Albufera silts/Fluvisol-Anthrosol	*Oryza sativa*
S28	30 S 0726234 - 4350558	Sollana	IP	Alluvial silt deposits/Anthrosol	*Phoenix canariensis*
S29	30 S 0724863 - 4359886	Silla	A	Black Albufera silts/Fluvisol-Anthrosol	*Oryza sativa*
S30	30 S 0725639 - 4365231	Massanassa	A	Black Albufera silts/Fluvisol-Anthrosol	*Oryza sativa*
S31	30 S 0722998 - 4374210	València	A	Alluvial silt deposits/Fluvisol-Anthrosol	*Malva sylvestris*
S32	30 S 0724645 - 4372714	València	UP	Alluvial silt deposits/Fluvisol	*Ficus sp.*
S33	30 S 0725199- 4371793	València	UP	Alluvial silt deposits/Fluvisol	*Ficus sp.*
S34	30 S 0726451- 4371973	València	UP	Alluvial silt deposits/Fluvisol	*Ficus sp.*
S35	30 S 0728393 - 4370558	València	UP	Alluvial silt deposits/Fluvisol	*Ficus sp.*

Table S1. (Continuation)

Samples	Coordinates (UTM)	Municipality	Land use	Parent material/Possible Reference soil group (WRB, 2022)	Plant sample
S36	30 S 0726796 - 4372185	València	UP	Alluvial silt deposits/Fluvisol	*Ficus sp.*
S37	30 S 0724963 - 4373348	València	UP	Alluvial silt deposits/Fluvisol	*Ficus sp.*
S38	30 S 0723342 - 4372661	València	UP	Alluvial silt deposits/Fluvisol	*Ficus sp.*
S39	30 S 0722749 - 4373294	València	UP	Alluvial silt deposits/Fluvisol	-
S40	30 S 0726935 - 4373145	València	UP	Alluvial silt deposits/Fluvisol	*Laurus nobilis*
S41	30 S 0729002 - 4372422	València	UP	Alluvial silt deposits/Fluvisol	*Ficus sp.*
S42	30 S 0726272 - 4372521	València	UP	Alluvial silt deposits/Fluvisol	*Ficus sp.*
S43	30 S 0728490 - 4370993	València	UP	Alluvial silt deposits/Fluvisol	*Ficus sp.*
S44	30 S 0727709- 4374389	Alboraia	A	Alluvial silt deposits/Fluvisol	*Citrus sp.*
S45	30 S 0724046 - 4369441	València	A	Alluvial silt deposits/Fluvisol	*Malva sylvestris*
S46	30 S 0729359 - 4361742	Albal	A	Black Albufera silts/Fluvisol-Anthrosol	*Oryza sativa*
S47	30 S 0725468 - 4362557	Albal	A	Black Albufera silts/Fluvisol	*na*
S48	30 S 0727854 - 4351216	Sollana	A	Black Albufera silts/Fluvisol	*Oryza sativa*
S49	30 S 0729807 - 4353833	Sueca	A	Black Albufera silts/Fluvisol	*Oryza sativa*
S50	30 S 0735597 - 4343725	Sueca	A	Black Albufera silts/Fluvisol	*Oryza sativa*
S51	30 S 0730499 - 4346229	Sueca	A	Black Albufera silts/Fluvisol	*Oryza sativa*
S52	30 S 0723728 - 4367037	Benetússer	UP	Alluvial silt deposits/Fluvisol	na
S53	30 S 0725805 - 4351198	Sollana	UP	Alluvial silt deposits/Fluvisol	na
S54	30 S 0723281 - 4364881	Catarroja	UP	Alluvial silt deposits/Fluvisol	na
S55	30 S 0732102 - 4342725	Sueca	UP	Alluvial silt deposits/Fluvisol	na

S, sample; A, agriculture; UP, urban park; IP, industrial park; F, forestry; na, data not available.

Table S2. Interpretation of the contamination indices.

Index	Range		Reference
ZnEq	≤ 300 mg Kg^{-1}	the soil does not present potential toxicity risks	Modified of Chumbley (1971)
	> 300 mg Kg^{-1}	the soil may pose potential toxicity risks	
Igeo	Class 0: Igeo ≤0	practically uncontaminated	Bhuiyan et al. (2010); Tamim et al. (2016); Gil et al. (2018)
	Class 1: 0 < Igeo < 1	uncontaminated to moderately contaminated	
	Class 2: 0 < Igeo < 2	moderately contaminated	
	Class 3: 2 < Igeo < 3	moderately to heavily contaminated	
	Class 4: 3 < Igeo < 4	heavily contaminated	
	Class 5: 4 < Igeo < 5	heavily to extremely contaminated	
	Class 6: 5 < Igeo	extremely contaminated	
EF	< 1.0	suggest a possible mobilization or depletion of metals	Tamim et al. (2016); Sappa et al. (2020)
	> 1.0	anthropogenic origin	
	1.5- 3.0	minor anthropogenic origin	
	3.0- 5.0	moderate anthropogenic origin	
	5.0- 10.0	severe anthropogenic origin	
	>10	very severe anthropogenic origin	
PLI	≤ 1	No HEs pollution	Tamim et al. (2016); Vineethkumar et al. (2020)
	> 1	HEs pollution exists	
mCd	mCd < 1.5	Nil to a very low degree of contamination	Vineethkumar et al. (2020)
	1.5 ≤ mCd < 2	Low degree of contamination	
	2 ≤ mCd < 4	A moderate degree of contamination	
	4 ≤ mCd < 8	A high degree of contamination	
	8 ≤ mCd < 16	A very high degree of contamination	
	16 ≤ mCd < 32	An extremely high degree of contamination	
	mCd ≤ 32	Ultrahigh degree of contamination	

ZnEq: Zinc equivalent; Igeo: Geoaccumulation index; EF: Enrichment factor; PLI: Pollution load index; mCd: modified degree of contamination.

Table S3. Soil characteristics.

Sample	Clay (%)	Silt (%)	Sand (%)	pH (H_2O)	EC (dS m^{-1} 25 °C)	$CaCO_3$ (%)	SOM (%)	N (%)	CEC ($cmol_c$ kg^{-1})	Land Use
S1	40.0	43.3	16.7	8.42 ± 0.05	0.23 ± 0.02	27.1 ± 1.2	2.67 ± 0.08	0.16 ± 0.06	18.97 ± 0.32	A
S2	39.1	23.6	37.3	8.45 ± 0.03	0.14 ± 0.01	5.9 ± 0.1	2.00 ± 0.08	0.12 ± 0.03	18.40 ± 0.06	A
S3	35.8	26.2	38.0	8.39 ± 0.02	0.16 ± 0.01	22.9 ± 2.2	3.14 ± 0.28	0.14 ± 0.10	18.60 ± 0.26	IP
S4	27.3	34.6	38.1	8.46 ± 0.11	0.20 ± 0.01	32.2 ± 1.9	3.21 ± 0.15	0.12 ± 0.02	16.50 ± 0.78	UP
S5	36.6	29.6	33.8	8.47 ± 0.01	0.13 ± 0.01	13.7 ± 0.6	2.20 ± 0.20	0.10 ± 0.07	17.20 ± 0.99	A
S6	18.4	64.9	16.7	8.41 ± 0.08	0.20 ± 0.00	36.3 ± 3.3	4.77 ± 0.09	0.14 ± 0.04	15.20 ± 2.84	UP
S7	20.2	20.1	59.7	8.29 ± 0.04	0.18 ± 0.00	25.3 ± 0.8	6.02 ± 0.51	0.29 ± 0.16	17.70 ± 0.98	IP
S8	22.1	26.3	51.6	8.30 ± 0.04	0.23 ± 0.01	23.4 ± 0.0	3.90 ± 0.25	0.17 ± 0.06	14.50 ± 2.37	UP
S9	27.1	32.3	40.6	8.61 ± 0.05	0.18 ± 0.01	30.0 ± 1.0	2.81 ± 0.27	0.13 ± 0.10	17.60 ± 0.85	A
S10	33.4	39.7	26.9	8.47 ± 0.14	0.17 ± 0.00	34.7 ± 1.1	2.57 ± 0.25	0.10 ± 0.10	17.40 ± 0.53	UP
S11	37.3	42.2	20.5	8.55 ± 0.05	0.20 ± 0.02	33.6 ± 3.3	3.07 ± 0.28	0.16 ± 0.08	19.90 ± 0.25	UP
S12	28.6	34.8	36.6	8.35 ± 0.05	0.21 ± 0.01	31.4 ± 2.9	4.77 ± 0.24	0.18 ± 0.04	16.60 ± 1.07	IP
S13	34.9	35.8	29.3	8.65 ± 0.09	0.31 ± 0.03	36.4 ± 1.2	2.72 ± 0.17	0.16 ± 0.00	22.20 ± 3.71	A
S14	21.3	20.9	57.8	7.37 ± 0.11	0.29 ± 0.01	35.3 ± 2.3	11.73 ± 0.47	0.59 ± 0.08	26.00 ± 3.77	IP
S15	29.2	26.1	44.7	8.46 ± 0.04	0.26 ± 0.02	14.6 ± 1.4	4.37 ± 0.03	0.27 ± 0.17	22.20 ± 2.95	IP
S16	29.6	29.4	41.0	8.48 ± 0.08	0.13 ± 0.01	17.1 ± 1.1	2.65 ± 0.26	0.13 ± 0.09	16.50 ± 0.73	A
S17	31.9	38.6	29.5	8.42 ± 0.1	0.16 ± 0.01	18.7 ± 1.6	2.54 ± 0.23	0.15 ± 0.05	22.00 ± 3.80	A
S18	34.3	50.4	15.3	8.51 ± 0.15	0.16 ± 0.01	36.5 ± 2.9	2.19 ± 0.08	0.13 ± 0.04	22.40 ± 4.52	A
S19	29.0	37.7	33.3	8.21 ± 0.08	0.19 ± 0.02	36.8 ± 3.4	2.49 ± 0.24	0.08 ± 0.11	16.20 ± 0.65	UP
S20	38.9	48.9	12.2	8.10 ± 0.08	0.19 ± 0.00	30.9 ± 3.0	4.31 ± 0.41	0.14 ± 0.19	22.80 ± 2.91	UP
S21	1.0	2.7	96.3	8.54 ± 0.12	0.11 ± 0.02	19.2 ± 1.0	1.19 ± 0.34	0.07 ± 0.19	3.00 ± 0.19	F
S22	4.6	5.2	90.2	7.94 ± 0.06	0.43 ± 0.02	28.9 ± 2.1	3.99 ± 0.26	0.32 ± 0.04	18.90 ± 1.08	A
S23	41.0	53.0	6.0	7.92 ± 0.23	0.78 ± 0.02	35.9 ± 3.1	5.28 ± 0.22	0.31 ± 0.07	23.70 ± 1.52	A
S24	40.0	55.0	5.0	8.00 ± 0.05	0.43 ± 0.00	36.3 ± 2.9	3.73 ± 0.07	0.21 ± 0.10	21.10 ± 0.02	A
S25	42.3	39.6	18.1	8.31 ± 0.04	0.19 ± 0.00	31.3 ± 3.0	4.02 ± 0.10	0.14 ± 0.03	21.00 ± 1.55	A
S26	44.0	49.5	6.5	8.01 ± 0.04	0.50 ± 0.00	35.5 ± 3	4.39 ± 0.08	0.23 ± 0.11	17.90 ± 2.70	A
S27	42.0	50.0	8.0	8.15 ± 0.11	0.28 ± 0.01	36.1 ± 2.8	3.47 ± 0.14	0.14 ± 0.00	20.20 ± 1.38	A
S28	47.9	35.3	16.8	8.19 ± 0.08	0.15 ± 0.01	35.6 ± 2.5	3.14 ± 0.30	0.10 ± 0.14	18.70 ± 2.11	IP
S29	29.0	43.0	28.0	8.00 ± 0.15	1.50 ± 0.05	28.5 ± 4.1	7.73 ± 0.65	0.38 ± 0.19	22.10 ± 0.49	A
S30	26.0	56.0	18.0	7.95 ± 0.11	0.63 ± 0.02	26.3 ± 4.3	4.82 ± 0.45	0.24 ± 0.15	21.80 ± 0.22	A
S31	22.7	27.2	50.1	7.84 ± 0.02	0.19 ± 0.01	30.8 ± 0.8	1.92 ± 0.03	0.14 ± 0.08	16.40 ± 2.51	A
S32	31.7	34.4	33.9	7.83 ± 0.01	0.26 ± 0.04	30.2 ± 0.7	2.06 ± 0.04	0.17 ± 0.09	20.50 ± 3.66	UP
S33	31.5	30.5	38.0	7.64 ± 0.01	0.46 ± 0.01	33.5 ± 0.9	2.98 ± 0.44	0.19 ± 0.18	20.90 ± 3.24	UP
S34	28.2	33.8	38.0	7.70 ± 0.04	0.31 ± 0.00	39.8 ± 1.1	2.36 ± 0.24	0.16 ± 0.06	18.60 ± 2.60	UP
S35	22.9	31.2	45.9	7.45 ± 0.09	0.37 ± 0.00	27.3 ± 0.7	7.06 ± 0.08	0.48 ± 0.28	21.00 ± 3.48	UP
S36	20.0	32.3	47.7	7.47 ± 0.13	0.41 ± 0.01	34.4 ± 0.9	5.60 ± 0.32	0.39 ± 0.05	24.80 ± 3.18	UP
S37	21.1	35.2	43.7	7.59 ± 0.03	0.42 ± 0.03	34.3 ± 0.8	5.56 ± 0.22	0.35 ± 0.09	23.40 ± 2.08	UP
S38	24.8	35.5	39.6	7.71 ± 0.02	0.24 ± 0.00	31.5 ± 0.7	3.59 ± 0.10	0.23 ± 0.09	23.40 ± 2.47	UP
S39	20.6	37.4	42.0	7.68 ± 0.04	0.55 ± 0.03	28.8 ± 0.8	4.02 ± 0.19	0.23 ± 0.03	19.90 ± 2.33	UP
S40	19.1	37.9	43.0	7.77 ± 0.08	0.19 ± 0.01	33.1 ± 0.9	4.63 ± 0.07	0.32 ± 0.18	21.60 ± 2.52	UP
S41	24.8	35.4	39.8	7.37 ± 0.06	0.37 ± 0.01	30.8 ± 0.8	5.10 ± 0.69	0.36 ± 0.23	22.64 ± 2.67	UP
S42	17.8	33.5	48.7	7.42 ± 0.01	1.08 ± 0.02	34.7 ± 1.0	4.34 ± 0.41	0.28 ± 0.09	23.20 ± 3.29	UP

Table S3. Soil characteristics (continuation)

Sample	Clay (%)	Silt (%)	Sand (%)	pH (H_2O)	EC (dS m^{-1} 25 °C)	$CaCO_3$ (%)	SOM (%)	N (%)	CEC ($cmol_c$ kg^{-1})	Land Use
S43	20.5	34.7	44.8	7.59 ± 0.02	0.21 ± 0.01	29.0 ± 0.7	4.91 ± 0.52	0.28 ± 0.17	23.10 ± 3.09	UP
S44	24.0	39.2	36.8	8.17 ± 0.06	0.16 ± 0.03	30.7 ± 0.8	2.88 ± 0.03	0.19 ± 0.11	19.10 ± 2.83	A
S45	28.9	36.3	34.8	7.91 ± 0.1	0.17 ± 0.00	29.6 ± 0.7	2.39 ± 0.28	0.18 ± 0.07	19.10 ± 2.76	A
S46	31.6	50.5	17.9	7.63 ± 0.23	2.10 ± 0.95	32.2 ± 3.3	6.16 ± 1.15	0.42 ± 0.08	19.20 ± 1.10	A
S47	29.0	53.5	17.6	7.63 ± 0.22	1.44 ± 0.42	34.3 ± 6.1	5.39 ± 1.14	0.40 ± 0.04	17.98 ± 1.20	A
S48	41.4	46.7	11.9	7.71 ± 0.24	1.31 ± 0.63	38.4 ± 1.3	3.99 ± 0.40	0.29 ± 0.04	20.55 ± 1.20	A
S49	31.7	56.0	12.4	7.89 ± 0.22	1.62 ± 0.64	47.1 ± 6.7	4.11 ± 0.86	0.29 ± 0.04	19.50 ± 1.30	A
S50	36.2	54.1	9.8	7.79 ± 0.08	1.39 ± 0.64	43.6 ± 2.9	4.65 ± 0.56	0.31 ± 0.03	18.90 ± 1.00	A
S51	41.0	37.5	21.5	7.73 ± 0.27	1.36 ± 0.99	38.8 ± 7.7	4.60 ± 0.26	0.37 ± 0.08	18.10 ± 3.30	A
S52	45.3	33.7	21.0	8.06 ± 0.13	0.47 ± 0.01	35.5 ± 3.8	1.29 ± 0.18	0.14 ± 0.01	17.43 ± 2.00	UP
S53	46.9	29.8	23.4	8.01 ± 0.06	0.35 ± 0.06	32.8 ± 3.8	3.13 ± 0.07	0.28 ± 0.03	19.00 ± 1.50	UP
S54	35.6	28.1	36.3	7.97 ± 0.14	0.52 ± 0.18	26.0 ± 2.0	4.61 ± 2.93	0.31 ± 0.14	20.28 ± 2.40	UP
S55	37.2	33.4	29.4	8.13 ± 0.31	0.43 ± 0.13	35.0 ± 4.5	3.24 ± 1.05	0.22 ± 0.16	19.10 ± 3.00	UP
**All soils except S21 and S22**										
MIN	17.8	20.1	5.0	7.37	0.13	5.9	1.29	0.08	14.50	
MAX	47.9	64.9	59.7	8.65	2.10	47.1	11.73	0.59	26.00	
AM	31.4	38.2	30.5	8.03	0.47	31.1	3.99	0.23	19.87	
STD	8.2	10.0	14.2	0.36	0.46	7.3	1.70	0.11	2.56	
CV	26.2	26.3	46.7	4.54	99.2	23.5	43.7	47.4	12.87	
**Agricultural soils (A)**										
MIN	22.70	23.60	5.00	7.63	0.13	5.9	1.92	0.10	16.40	
MAX	44.00	56.00	50.10	8.65	2.10	47.1	7.73	0.42	23.70	
AM	34.35	42.92	22.75	8.11	0.65	30.9	3.72	0.22	19.66	
STD	6.40	10.02	12.94	0.33	0.62	9.4	1.47	0.10	2.02	
CV	18.65	23.34	56.88	4.02	95.8	30.4	39.51	44.57	10.28	
**Urban park soils (UP)**										
MIN	17.80	26.30	12.20	7.37	0.17	23.4	1.29	0.08	14.50	
MAX	46.85	64.90	51.60	8.55	1.08	39.8	7.06	0.48	24.80	
AM	28.45	36.13	35.41	7.91	3.49	32.4	3.86	2.31	20.06	
STD	8.62	7.85	10.86	0.37	0.20	3.8	1.34	0.10	2.88	
CV	30.29	21.72	30.67	4.68	5.72	11.6	34.79	4.44	14.36	
**Industrial park soils (IP)**										
MIN	20.20	20.10	16.80	7.37	0.15	14.6	3.14	0.10	16.60	
MAX	47.90	35.30	59.70	8.46	0.29	35.6	11.73	0.59	26.00	
AM	30.50	27.23	42.27	8.18	2.08	27.5	5.53	2.61	19.97	
STD	10.27	6.57	15.81	0.40	0.06	8.2	3.23	0.18	3.50	
CV	33.66	24.12	37.41	4.95	2.71	29.7	58.35	6.76	17.54	

Mean values ± standard deviation; MIN, minimum value; MAX, maximum value; AM, mean values; CV, coefficient of variation; EC, electrical conductivity; CEC, cationic exchange; SOM, soil organic matter; N, total nitrogen.

Table S4. Soil element concentrations.

Sample	Al[a]	As	Cd	Co	Cr	Cu	Fe[a]	Li	Land use
1	3.89 ± 0.17	13.95 ± 0.48	0.81 ± 0.30	12.84 ± 0.49	45.18 ± 16.49	32.43 ± 1.81	2.18 ± 0.48	38.23 ± 0.55	A
2	4.19 ± 0.18	14.22 ± 0.49	0.89 ± 0.33	14.60 ± 0.56	48.44 ± 17.68	32.99 ± 1.84	2.33 ± 0.51	30.31 ± 0.44	A
3	3.20 ± 0.14	12.56 ± 0.43	0.69 ± 0.25	10.32 ± 0.39	33.69 ± 12.30	19.04 ± 1.06	1.87 ± 0.41	29.57 ± 0.43	IP
4	3.03 ± 0.13	8.43 ± 0.29	0.51 ± 0.19	10.24 ± 0.39	41.35 ± 15.09	16.43 ± 0.92	1.59 ± 0.35	26.66 ± 0.39	UP
5	3.62 ± 0.16	11.42 ± 0.39	0.67 ± 0.24	12.02 ± 0.46	38.76 ± 14.15	13.29 ± 0.74	1.95 ± 0.43	27.96 ± 0.41	A
6	1.02 ± 0.04	4.74 ± 0.16	0.25 ± 0.09	4.64 ± 0.18	14.41 ± 5.26	22.04 ± 1.23	0.56 ± 0.12	9.90 ± 0.14	UP
7	2.00 ± 0.09	5.63 ± 0.19	0.39 ± 0.14	7.53 ± 0.29	28.80 ± 10.51	9.63 ± 0.54	1.14 ± 0.25	16.89 ± 0.24	IP
8	1.69 ± 0.07	4.87 ± 0.17	0.32 ± 0.12	6.37 ± 0.24	21.35 ± 7.79	10.23 ± 0.57	1.00 ± 0.22	15.63 ± 0.23	UP
9	2.38 ± 0.10	9.98 ± 0.34	0.68 ± 0.25	8.96 ± 0.34	60.12 ± 21.94	33.71 ± 1.88	1.43 ± 0.32	22.94 ± 0.33	A
10	3.49 ± 0.15	9.05 ± 0.31	0.61 ± 0.22	11.66 ± 0.45	40.33 ± 14.72	25.85 ± 1.44	1.81 ± 0.40	32.89 ± 0.48	UP
11	3.45 ± 0.15	11.81 ± 0.40	0.78 ± 0.29	11.41 ± 0.44	57.90 ± 21.13	36.72 ± 2.05	1.78 ± 0.39	34.98 ± 0.51	UP
12	2.77 ± 0.12	7.43 ± 0.25	0.87 ± 0.32	9.76 ± 0.37	33.25 ± 12.14	28.13 ± 1.57	1.49 ± 0.33	25.53 ± 0.37	IP
13	3.60 ± 0.16	10.65 ± 0.36	0.74 ± 0.27	11.41 ± 0.44	53.65 ± 19.58	32.03 ± 1.79	1.81 ± 0.40	34.42 ± 0.50	A
14	2.44 ± 0.11	5.78 ± 0.20	0.50 ± 0.19	8.79 ± 0.34	34.65 ± 12.65	20.53 ± 1.15	1.32 ± 0.29	19.61 ± 0.28	IP
15	3.78 ± 0.16	10.23 ± 0.35	0.59 ± 0.22	10.90 ± 0.42	43.93 ± 16.04	11.43 ± 0.64	1.99 ± 0.44	30.07 ± 0.44	IP
16	3.43 ± 0.15	9.43 ± 0.32	0.63 ± 0.23	11.55 ± 0.44	39.70 ± 14.49	14.49 ± 0.81	1.84 ± 0.41	26.51 ± 0.38	A
17	3.36 ± 0.15	10.20 ± 0.35	0.68 ± 0.25	11.21 ± 0.43	38.30 ± 13.98	31.03 ± 1.73	1.86 ± 0.41	30.66 ± 0.44	A
18	3.44 ± 0.15	8.67 ± 0.30	0.64 ± 0.24	11.69 ± 0.45	39.16 ± 14.29	24.65 ± 1.38	1.86 ± 0.41	33.93 ± 0.49	A
19	2.61 ± 0.11	6.92 ± 0.24	0.43 ± 0.16	9.29 ± 0.35	30.49 ± 11.13	10.69 ± 0.60	1.43 ± 0.32	23.58 ± 0.34	UP
20	3.10 ± 0.13	4.99 ± 0.17	0.51 ± 0.19	10.85 ± 0.41	34.09 ± 12.44	16.58 ± 0.93	1.68 ± 0.37	29.08 ± 0.42	UP
21	0.34 ± 0.01	2.89 ± 0.10	0.12 ± 0.04	0.71 ± 0.03	4.28 ± 1.56	1.02 ± 0.06	0.24 ± 0.05	4.04 ± 0.06	F
22	0.51 ± 0.02	4.75 ± 0.16	0.29 ± 0.10	2.66 ± 0.10	9.36 ± 3.42	12.25 ± 0.68	0.44 ± 0.10	6.95 ± 0.10	A
23	3.34 ± 0.22	10.26 ± 0.41	1.35 ± 0.99	<0.01	33.61 ± 0.78	40.52 ± 0.20	1.83 ± 0.81	31.87 ± 7.84	A
24	4.42 ± 0.05	13.62 ± 3.90	1.05 ± 0.54	<0.01	41.20 ± 2.41	40.49 ± 1.70	2.50 ± 0.44	41.18 ± 2.30	A
25	3.55 ± 0.38	10.45 ± 4.32	0.60 ± 0.08	<0.01	34.96 ± 0.24	17.23 ± 0.62	2.23 ± 0.48	32.91 ± 7.51	A
26	3.95 ± 0.08	12.13 ± 2.43	0.68 ± 0.03	<0.01	38.68 ± 0.33	33.47 ± 0.07	2.31 ± 0.30	38.70 ± 1.02	A
27	4.30 ± 0.02	13.07 ± 7.05	0.77 ± 0.02	<0.01	42.74 ± 0.05	46.53 ± 0.86	2.61 ± 1.66	42.53 ± 1.82	A
28	4.02 ± 0.67	12.47 ± 5.91	0.92 ± 0.08	<0.01	39.09 ± 2.78	22.59 ± 0.53	2.67 ± 2.89	38.02 ± 5.64	IP
29	3.80 ± 0.16	7.32 ± 0.25	0.68 ± 0.25	10.12 ± 0.39	51.92 ± 18.95	20.77 ± 1.16	1.74 ± 0.39	34.82 ± 0.50	A
30	2.78 ± 0.28	9.12 ± 3.67	1.15 ± 0.46	<0.01	46.96 ± 3.56	32.80 ± 1.19	1.80 ± 1.18	28.21 ± 10.07	A
31	2.27 ± 0.10	7.72 ± 0.26	0.50 ± 0.18	8.35 ± 0.32	27.92 ± 10.19	30.66 ± 1.71	1.31 ± 0.29	25.24 ± 0.37	A
32	2.74 ± 0.12	8.93 ± 0.31	0.52 ± 0.19	10.28 ± 0.39	37.82 ± 13.80	31.19 ± 1.74	1.56 ± 0.35	28.49 ± 0.41	UP
33	2.69 ± 0.12	9.40 ± 0.32	0.63 ± 0.23	9.99 ± 0.38	50.10 ± 18.29	77.51 ± 4.33	1.66 ± 0.37	28.51 ± 0.41	UP
34	2.81 ± 0.12	8.02 ± 0.27	0.51 ± 0.19	9.94 ± 0.38	39.41 ± 14.39	32.17 ± 1.79	1.56 ± 0.35	29.39 ± 0.43	UP
35	2.46 ± 0.11	8.30 ± 0.28	0.52 ± 0.19	8.85 ± 0.34	38.53 ± 14.06	33.40 ± 1.86	1.40 ± 0.31	25.48 ± 0.37	UP
36	1.82 ± 0.08	5.77 ± 0.20	0.45 ± 0.16	7.13 ± 0.27	44.26 ± 16.15	35.40 ± 1.98	1.10 ± 0.24	21.12 ± 0.31	UP
37	2.35 ± 0.10	6.85 ± 0.23	0.57 ± 0.21	8.16 ± 0.31	61.19 ± 22.33	47.95 ± 2.68	1.34 ± 0.30	25.10 ± 0.36	UP
38	2.53 ± 0.11	7.17 ± 0.25	0.45 ± 0.16	9.24 ± 0.35	32.76 ± 11.96	19.08 ± 1.06	1.44 ± 0.32	26.22 ± 0.38	UP
39	2.03 ± 0.09	6.82 ± 0.23	0.40 ± 0.15	7.66 ± 0.29	25.06 ± 9.15	20.92 ± 1.17	1.13 ± 0.25	22.93 ± 0.33	UP
40	1.92 ± 0.08	7.60 ± 0.26	0.85 ± 0.31	8.07 ± 0.31	55.79 ± 20.36	54.97 ± 3.07	1.26 ± 0.28	22.09 ± 0.32	UP
41	2.52 ± 0.11	7.71 ± 0.26	0.50 ± 0.18	8.96 ± 0.34	42.44 ± 15.49	31.22 ± 1.74	1.47 ± 0.32	28.41 ± 0.41	UP
42	1.64 ± 0.07	7.96 ± 0.27	0.57 ± 0.21	7.42 ± 0.28	41.33 ± 15.09	91.10 ± 5.08	1.15 ± 0.25	19.57 ± 0.28	UP
43	2.26 ± 0.10	8.10 ± 0.28	0.47 ± 0.17	9.13 ± 0.35	64.65 ± 23.60	30.36 ± 1.69	1.40 ± 0.31	23.81 ± 0.35	UP
44	2.78 ± 0.12	9.90 ± 0.34	0.76 ± 0.28	11.17 ± 0.43	55.75 ± 20.35	141.73 ± 7.91	1.68 ± 0.37	31.66 ± 0.46	A

45	3.14 ± 0.14	12.32 ± 0.42	0.83 ± 0.31	11.04 ± 0.42	110.26 ± 40.25	66.01 ± 3.68	1.87 ± 0.41	34.84 ± 0.51	A
46	4.36 ± 0.27	3.48 ± 2.02	0.65 ± 0.03	8.80 ± 0.46	67.60 ± 14.64	47.81 ± 11.54	2.15 ± 0.10	57.50 ± 1.98	A
47	4.29 ± 0.59	2.34 ± 1.65	0.84 ± 0.24	8.81 ± 1.00	65.32 ± 13.41	48.62 ± 13.92	2.17 ± 0.29	53.41 ± 3.57	A
48	3.93 ± 0.17	0.67 ± 0.02	0.75 ± 0.27	8.82 ± 0.34	45.85 ± 16.74	20.43 ± 1.14	1.99 ± 0.44	49.72 ± 0.72	A
49	4.24 ± 0.31	3.30 ± 1.47	0.63 ± 0.03	9.16 ± 0.88	48.55 ± 6.45	19.38 ± 2.10	2.20 ± 0.13	52.96 ± 3.61	A
50	4.45 ± 0.56	1.02 ± 0.31	0.74 ± 0.06	9.94 ± 1.24	51.42 ± 5.94	22.28 ± 2.47	2.21 ± 0.31	54.25 ± 5.38	A
51	3.53 ± 0.15	<0.01	0.54 ± 0.20	7.29 ± 0.28	44.80 ± 16.35	31.77 ± 1.77	1.75 ± 0.39	45.08 ± 0.65	A
52	2.83 ± 0.38	0.96 ± 0.56	0.52 ± 0.04	6.18 ± 1.03	35.13 ± 3.07	28.13 ± 5.30	1.32 ± 0.26	32.65 ± 3.79	UP
53	3.18 ± 0.19	0.56 ± 0.02	0.58 ± 0.07	7.42 ± 0.25	36.03 ± 1.79	18.44 ± 1.98	1.52 ± 0.01	35.69 ± 0.79	UP
54	3.19 ± 0.16	1.63 ± 0.95	0.53 ± 0.04	6.71 ± 0.26	43.69 ± 6.13	23.19 ± 0.49	1.46 ± 0.19	35.23 ± 0.14	UP
55	2.80 ± 0.36	<0.01	0.40 ± 0.00	6.10 ± 0.56	37.84 ± 1.97	18.91 ± 1.29	1.36 ± 0.23	35.14 ± 3.01	UP
**All soils**									
MIN	0.34	<0.01	0.12	<0.01	4.28	1.02	0.24	4.04	
MAX	4.45	14.22	1.35	14.60	110.26	141.73	2.67	57.50	
AM	2.99	7.80	0.63	9.05	42.25	31.49	1.65	30.60	
STD	0.95	3.63	0.22	2.49	15.74	22.30	0.49	10.88	
CV	31.72	46.52	34.53	27.56	37.25	70.82	29.99	35.56	
**Agricultural soils (A)**									
MIN	0.51	<0.01	0.29	<0.01	9.36	12.25	0.44	6.95	
MAX	4.45	14.22	1.35	14.60	110.26	141.73	2.61	57.50	
AM	3.50	8.67	0.74	9.95	46.94	35.64	1.93	36.35	
STD	0.87	4.11	0.21	2.52	17.79	25.51	0.44	11.54	
CV	25.01	47.36	28.55	25.35	37.90	71.57	22.58	31.76	
**Urban park soils (UP)**									
MIN	1.02	<0.01	0.25	4.64	14.41	10.23	0.56	9.90	
MAX	3.49	11.81	0.85	11.66	64.65	91.10	1.81	35.69	
AM	2.50	6.59	0.53	8.43	39.95	31.95	1.38	26.31	
STD	0.59	2.75	0.15	1.74	12.19	19.82	0.26	6.37	
CV	23.61	41.77	28.14	20.70	30.52	62.05	19.16	24.22	
**Industrial park soils (IP)**									
MIN	2.00	5.63	0.39	<0.01	28.80	9.63	1.14	16.89	
MAX	4.02	12.56	0.92	10.90	43.93	28.13	2.67	38.02	
AM	3.03	9.02	0.66	9.46	35.57	18.56	1.75	26.62	
STD	0.78	3.17	0.21	1.33	5.25	6.97	0.55	7.69	
CV	25.72	35.21	31.38	14.06	14.77	37.54	31.76	28.88	

Mean values ± standard deviation; MIN, minimum value; MAX, maximum value; AM, mean values; elements in mg kg^{-1} except elements[a] in g / 100 g

Table S4. Soil element concentrations (continuation).

Sample	Mg[a]	Mn	Ni	Pb	Sr	V	Zn	Land use
1	1.13 ± 0.01	395.65 ± 29.67	20.66 ± 2.67	80.51 ± 1.98	225.81 ± 35.50	42.47 ± 0.82	92.46 ± 4.18	A
2	0.47 ± 0.04	357.54 ± 26.82	18.18 ± 2.35	45.82 ± 1.13	87.26 ± 13.72	50.27 ± 0.97	86.41 ± 3.91	A
3	0.72 ± 0.07	354.43 ± 26.58	13.92 ± 1.80	42.96 ± 1.06	123.26 ± 19.38	35.44 ± 0.68	58.39 ± 2.64	IP
4	0.58 ± 0.06	293.12 ± 21.98	12.77 ± 1.65	29.81 ± 0.73	178.30 ± 28.03	35.32 ± 0.68	47.79 ± 2.16	UP
5	0.46 ± 0.04	309.94 ± 23.25	15.60 ± 2.01	56.04 ± 1.38	80.50 ± 12.65	42.67 ± 0.82	46.20 ± 2.09	A
6	0.30 ± 0.03	122.23 ± 9.17	5.71 ± 0.74	345.99 ± 8.51	98.01 ± 15.41	14.69 ± 0.28	41.49 ± 1.88	UP
7	0.35 ± 0.03	179.17 ± 13.44	9.26 ± 1.20	20.34 ± 0.50	102.09 ± 16.05	25.56 ± 0.49	44.38 ± 2.01	IP
8	0.42 ± 0.04	162.47 ± 12.19	8.15 ± 1.05	21.65 ± 0.53	121.03 ± 19.03	21.21 ± 0.41	35.11 ± 1.59	UP
9	0.65 ± 0.06	261.64 ± 19.62	17.08 ± 2.20	97.39 ± 2.40	195.28 ± 30.70	30.39 ± 0.59	94.10 ± 4.25	A
10	0.76 ± 0.07	321.40 ± 24.11	15.71 ± 2.03	40.38 ± 0.99	189.20 ± 29.74	41.11 ± 0.79	70.26 ± 3.18	UP
11	0.98 ± 0.09	341.36 ± 25.60	16.70 ± 2.16	58.15 ± 1.43	288.40 ± 45.34	38.58 ± 0.74	81.44 ± 3.68	UP
12	0.65 ± 0.06	261.89 ± 19.64	12.55 ± 1.62	36.21 ± 0.89	197.76 ± 31.09	33.33 ± 0.64	107.23 ± 4.85	IP
13	0.97 ± 0.09	356.00 ± 26.70	16.04 ± 2.07	59.61 ± 1.47	379.01 ± 39.58	39.20 ± 0.76	91.93 ± 4.16	A
14	0.53 ± 0.05	196.88 ± 14.77	11.85 ± 1.53	56.09 ± 1.38	134.85 ± 21.20	30.86 ± 0.60	138.48 ± 6.26	IP
15	0.51 ± 0.05	216.27 ± 16.22	15.13 ± 1.95	25.60 ± 0.63	98.80 ± 15.53	42.55 ± 0.82	48.27 ± 2.18	IP
16	0.58 ± 0.05	283.37 ± 21.25	14.56 ± 1.88	29.99 ± 0.74	151.26 ± 23.78	41.42 ± 0.80	54.80 ± 2.48	A
17	0.99 ± 0.09	368.91 ± 27.67	14.52 ± 1.87	33.42 ± 0.82	198.66 ± 31.23	40.51 ± 0.78	54.38 ± 2.46	A
18	2.22 ± 0.21	425.32 ± 31.90	16.21 ± 2.09	29.79 ± 0.73	332.10 ± 52.21	44.04 ± 0.85	49.29 ± 2.23	A
19	1.37 ± 0.13	269.29 ± 20.20	12.33 ± 1.59	19.66 ± 0.48	152.02 ± 23.90	35.01 ± 0.68	29.11 ± 1.32	UP
20	2.11 ± 0.20	402.04 ± 30.15	15.97 ± 2.06	20.33 ± 0.50	336.85 ± 52.95	39.49 ± 0.76	41.65 ± 1.88	UP
21	0.29 ± 0.03	137.23 ± 10.29	1.55 ± 0.20	9.53 ± 0.23	68.87 ± 5.83	5.80 ± 0.11	7.81 ± 0.35	F
22	0.58 ± 0.06	196.97 ± 14.77	2.64 ± 0.34	9.17 ± 0.23	175.85 ± 27.64	9.34 ± 0.18	53.53 ± 2.42	A
23	1.53 ± 0.09	262.70 ± 17.42	12.36 ± 8.32	21.41 ± 1.10	243.36 ± 48.76	36.16 ± 0.80	49.58 ± 1.31	A
24	1.92 ± 0.69	333.24 ± 15.20	12.90 ± 1.23	24.94 ± 9.10	128.49 ± 16.11	49.32 ± 2.42	44.62 ± 4.24	A
25	2.14 ± 0.35	371.61 ± 56.68	11.38 ± 2.67	22.91 ± 6.56	78.32 ± 26.84	37.92 ± 1.88	40.24 ± 3.57	A
26	1.89 ± 0.20	340.23 ± 9.89	12.56 ± 3.69	21.62 ± 0.78	231.36 ± 51.61	43.74 ± 0.50	39.53 ± 1.09	A
27	1.96 ± 0.18	338.92 ± 32.59	14.68 ± 1.76	21.29 ± 2.51	66.19 ± 19.50	47.56 ± 2.11	43.19 ± 2.01	A
28	1.08 ± 0.09	430.01 ± 70.07	16.37 ± 4.80	39.47 ± 2.29	170.55 ± 69.31	44.31 ± 3.76	109.99 ± 5.48	IP
29	1.11 ± 0.10	213.28 ± 16.00	16.76 ± 2.16	40.22 ± 0.99	393.40 ± 61.84	47.68 ± 0.92	59.96 ± 2.71	A
30	0.95 ± 0.56	268.47 ± 12.36	15.68 ± 1.92	42.61 ± 4.15	169.64 ± 35.30	30.18 ± 2.41	92.33 ± 4.05	A
31	1.01 ± 0.10	323.94 ± 24.30	10.25 ± 1.32	80.50 ± 1.98	228.87 ± 35.98	27.04 ± 0.52	73.23 ± 3.31	A
32	0.78 ± 0.07	313.24 ± 23.49	13.06 ± 1.69	65.29 ± 1.61	205.41 ± 32.29	33.19 ± 0.64	55.99 ± 2.53	UP
33	0.86 ± 0.08	342.92 ± 25.72	14.39 ± 1.86	121.20 ± 2.98	255.69 ± 40.19	32.81 ± 0.63	147.50 ± 6.67	UP
34	1.20 ± 0.11	283.75 ± 21.28	12.87 ± 1.66	59.45 ± 1.46	240.52 ± 37.81	35.07 ± 0.68	73.69 ± 3.33	UP
35	0.87 ± 0.08	294.18 ± 22.06	12.03 ± 1.55	63.92 ± 1.57	260.19 ± 40.90	29.37 ± 0.57	87.23 ± 3.94	UP
36	0.82 ± 0.08	251.96 ± 18.90	9.96 ± 1.29	51.92 ± 1.28	224.68 ± 35.32	22.67 ± 0.44	94.53 ± 4.27	UP
37	0.90 ± 0.09	287.00 ± 21.52	10.61 ± 1.37	59.82 ± 1.47	266.66 ± 41.92	27.57 ± 0.53	125.40 ± 5.67	UP
38	0.88 ± 0.08	307.92 ± 23.09	11.86 ± 1.53	28.36 ± 0.70	212.35 ± 33.38	29.51 ± 0.57	55.60 ± 2.51	UP
39	0.95 ± 0.09	269.60 ± 20.22	9.46 ± 1.22	34.50 ± 0.85	195.25 ± 30.69	24.37 ± 0.47	53.56 ± 2.42	UP
40	0.86 ± 0.08	387.26 ± 29.04	10.59 ± 1.37	155.50 ± 3.83	256.01 ± 40.24	24.73 ± 0.48	297.66 ± 13.45	UP
41	1.08 ± 0.10	304.96 ± 22.87	11.16 ± 1.44	59.87 ± 1.47	255.06 ± 40.09	28.73 ± 0.55	92.83 ± 4.20	UP
42	0.90 ± 0.09	271.55 ± 20.37	11.26 ± 1.45	177.56 ± 4.37	265.42 ± 41.72	25.01 ± 0.48	160.15 ± 7.24	UP
43	0.93 ± 0.09	309.23 ± 23.19	13.72 ± 1.77	46.27 ± 1.14	225.60 ± 35.47	28.54 ± 0.55	89.62 ± 4.05	UP

Table S4. Soil element concentrations (continuation).

Sample	Mg[a]			Mn			Ni			Pb			Sr			V			Zn			Land use
44	1.16	±	0.11	370.88	±	27.82	15.35	±	1.98	149.69	±	3.68	285.35	±	44.86	32.92	±	0.64	226.50	±	10.24	A
45	1.20	±	0.11	369.62	±	27.72	17.70	±	2.29	90.55	±	2.23	239.25	±	37.61	34.97	±	0.67	142.84	±	6.46	A
46	1.22	±	0.06	257.66	±	27.85	22.28	±	2.99	35.03	±	4.49	505.22	±	62.91	48.85	±	1.42	71.42	±	12.69	A
47	1.05	±	0.10	253.59	±	8.01	28.59	±	6.98	48.60	±	8.36	444.23	±	57.90	48.29	±	5.34	76.38	±	10.49	A
48	1.45	±	0.14	299.62	±	22.47	37.50	±	4.84	108.32	±	2.66	667.92	±	64.00	49.34	±	0.95	38.63	±	1.75	A
49	1.84	±	0.10	350.26	±	21.99	20.98	±	2.63	32.38	±	15.54	604.62	±	31.80	51.81	±	3.16	38.52	±	3.61	A
50	1.85	±	0.15	314.48	±	36.54	22.71	±	2.49	36.51	±	11.48	574.69	±	43.07	55.49	±	6.19	38.22	±	4.28	A
51	1.68	±	0.16	238.47	±	17.89	15.47	±	2.00	18.82	±	0.46	696.66	±	89.51	40.85	±	0.79	43.66	±	1.97	A
52	0.55	±	0.05	239.55	±	33.36	13.28	±	1.38	24.89	±	4.86	172.05	±	3.17	27.00	±	0.52	70.51	±	3.19	UP
53	0.95	±	0.05	279.66	±	5.01	21.92	±	9.05	108.85	±	2.68	338.33	±	1.51	34.71	±	0.67	58.70	±	2.65	UP
54	0.60	±	0.04	245.17	±	5.77	13.78	±	0.08	31.27	±	3.27	191.23	±	12.34	31.02	±	0.60	65.92	±	2.98	UP
55	1.56	±	0.25	279.15	±	17.24	12.54	±	1.35	37.90	±	11.36	315.96	±	37.46	29.47	±	0.57	52.37	±	2.37	UP
**All soils**																						
MIN	0.29			122.23			1.55			9.17			66.19			5.80			7.81			
MAX	2.22			430.01			37.50			345.99			696.66			55.49			297.66			
AM	1.04			294.86			14.42			56.91			245.89			35.08			76.08			
STD	0.50			68.48			5.58			54.06			144.52			10.34			48.91			
CV	48.33			23.22			38.70			94.99			58.77			29.48			64.28			
**Agricultural soils (A)**																						
MIN	0.46			196.97			2.64			9.17			66.19			9.34			38.22			
MAX	2.22			425.32			37.50			345.99			696.66			55.49			226.50			
AM	1.29			312.76			16.94			49.06			291.85			40.97			68.75			
STD	0.54			58.96			6.51			33.93			189.75			9.88			41.48			
CV	41.52			18.85			38.40			69.16			65.02			24.11			60.34			
**Urban park soils (UP)**																						
MIN	0.30			122.23			5.71			19.66			98.01			14.69			29.11			
MAX	2.11			402.04			21.92			345.99			338.33			41.11			297.66			
AM	0.92			283.46			12.46			72.10			228.38			29.63			85.44			
STD	0.38			60.53			3.15			73.28			62.38			5.88			57.61			
CV	41.80			21.35			25.26			101.63			27.31			19.86			67.43			
**Industrial park soils (IP)**																						
MIN	0.35			179.17			9.26			20.34			98.80			25.56			44.38			
MAX	1.08			430.01			16.37			56.09			197.76			44.31			138.48			
AM	0.64			273.11			13.18			36.78			137.88			35.34			84.46			
STD	0.25			99.22			2.53			12.76			39.20			7.10			39.20			
CV	39.03			36.33			19.21			34.68			28.43			20.09			46.42			

Mean values ± standard deviation; MIN, minimum value; MAX, maximum value; AM, mean values; elements in mg kg^{-1} except elements[a] in g / 100 g.

Table S5. Plant element concentrations.

Sample	Al			As			Cd			Co			Cr			Cu			Fe			Li			Land use
1	111.05	±	11.27	7.83	±	1.29	0.01	±	0.00	0.04	±	0.02	1.17	±	0.24	6.86	±	1.86	96.01	±	8.01	6.79	±	2.03	A
2	134.99	±	13.70	9.05	±	1.49	<0.01			0.06	±	0.02	1.37	±	0.28	4.66	±	1.26	130.20	±	10.86	3.88	±	1.16	A
3	304.70	±	30.93	5.91	±	0.97	0.10	±	0.01	0.23	±	0.09	3.86	±	0.77	7.32	±	1.99	337.27	±	18.13	2.86	±	0.85	IP
4	181.65	±	18.44	6.38	±	1.05	0.01	±	0.00	0.14	±	0.06	3.68	±	0.74	5.94	±	1.61	203.23	±	16.95	3.32	±	0.99	UP
5	322.85	±	32.77	8.13	±	1.34	0.03	±	0.00	0.10	±	0.04	2.89	±	0.58	5.77	±	1.57	237.62	±	19.82	3.23	±	0.97	A
6	216.59	±	21.98	5.38	±	0.89	0.02	±	0.00	0.14	±	0.06	2.06	±	0.41	6.78	±	1.84	217.67	±	18.15	2.10	±	0.63	UP
7	324.29	±	32.92	6.63	±	1.09	0.02	±	0.00	0.30	±	0.12	4.41	±	0.89	6.78	±	1.84	329.10	±	27.45	2.87	±	0.86	IP
8	173.12	±	17.57	6.41	±	1.06	0.01	±	0.00	0.10	±	0.04	2.31	±	0.46	7.79	±	2.11	198.00	±	16.51	3.65	±	1.09	UP
9	70.71	±	7.18	7.40	±	1.22	<0.01			0.13	±	0.05	3.62	±	0.73	8.27	±	2.25	77.48	±	6.46	7.79	±	2.33	A
10	116.22	±	11.80	5.79	±	0.95	0.03	±	0.00	0.15	±	0.06	1.38	±	0.28	5.55	±	1.51	138.38	±	11.54	2.95	±	0.88	UP
11	136.02	±	13.81	7.25	±	1.20	0.10	±	0.01	0.17	±	0.07	4.82	±	0.97	7.85	±	2.13	200.40	±	16.71	4.89	±	1.46	UP
12	80.97	±	8.22	5.32	±	0.88	0.05	±	0.00	0.06	±	0.02	2.26	±	0.45	8.28	±	2.25	143.13	±	11.94	2.74	±	0.82	IP
13	161.49	±	16.39	6.36	±	1.05	0.06	±	0.00	0.09	±	0.04	1.54	±	0.31	7.46	±	2.03	164.71	±	13.74	4.19	±	1.25	A
14	147.88	±	15.01	5.99	±	0.99	0.04	±	0.00	0.11	±	0.05	1.70	±	0.34	6.13	±	1.66	149.75	±	12.49	2.71	±	0.81	IP
16	146.16	±	14.84	8.04	±	1.33	0.01	±	0.00	0.06	±	0.02	0.84	±	0.17	6.31	±	1.71	126.84	±	10.58	4.16	±	1.24	A
17	130.91	±	13.29	6.74	±	1.11	0.01	±	0.00	0.13	±	0.05	1.88	±	0.38	6.64	±	1.80	112.20	±	9.36	2.63	±	0.79	A
18	107.09	±	10.87	6.24	±	1.03	<0.01			0.09	±	0.04	1.31	±	0.26	7.06	±	1.92	104.59	±	8.72	3.88	±	1.16	A
19	95.56	±	9.70	7.27	±	1.20	0.18	±	0.01	0.13	±	0.05	0.98	±	0.20	9.21	±	2.50	134.82	±	11.24	8.97	±	1.68	UP
20	241.93	±	24.56	6.60	±	1.09	0.07	±	0.00	0.18	±	0.07	3.38	±	0.68	16.51	±	3.48	409.21	±	34.13	2.49	±	0.75	UP
21	82.98	±	8.42	3.66	±	0.60	0.01	±	0.00	0.11	±	0.04	0.71	±	0.14	4.38	±	1.19	84.41	±	7.04	1.68	±	0.50	F
22	339.86	±	34.50	5.24	±	0.86	0.02	±	0.00	0.06	±	0.02	3.48	±	0.70	5.39	±	1.46	197.05	±	16.43	2.47	±	0.74	A
23	2115.94	±	114.77	16.30	±	2.69	0.45	±	0.03	0.48	±	0.19	9.28	±	1.86	6.69	±	1.82	1209.87	±	90.90	4.97	±	1.48	A
25	95.43	±	9.69	6.72	±	1.11	<0.01			0.05		0.02	1.48		0.30	9.89		2.68	80.41		6.71	4.86		1.45	A
26	1083.79	±	110.00	10.19	±	1.68	1.36	±	0.10	0.92	±	0.37	4.30	±	0.86	7.86	±	2.13	681.66	±	56.85	3.79	±	1.13	A
27	886.71	±	90.00	9.99	±	1.65	1.43	±	0.10	0.84	±	0.33	2.52	±	0.50	8.53	±	2.31	604.24	±	50.39	3.65	±	1.09	A
28	360.06	±	36.55	7.04	±	1.16	0.40	±	0.03	0.57	±	0.22	2.53	±	0.51	17.63	±	4.79	688.35	±	57.41	3.90	±	1.16	IP
29	2344.44	±	207.96	16.62	±	2.74	0.34	±	0.02	1.30	±	0.52	6.81	±	1.37	10.22	±	2.78	1352.95	±	112.84	5.85	±	1.75	A
30	3086.73	±	313.30	20.25	±	3.34	0.11	±	0.01	0.83	±	0.33	33.38	±	2.70	13.20	±	3.58	1959.97	±	103.46	6.00	±	1.79	A
31	364.22	±	36.97	7.35	±	1.21	0.11	±	0.01	0.27	±	0.11	1.93	±	0.39	23.47	±	2.37	401.31	±	33.47	2.97	±	0.89	A
32	150.52	±	15.28	6.10	±	1.01	0.01	±	0.00	0.14	±	0.06	2.52	±	0.50	16.24	±	4.41	377.92	±	31.52	2.17	±	0.65	UP
33	1447.57	±	146.93	3.92	±	0.65	<0.01			0.36	±	0.14	2.14	±	0.43	17.47	±	4.74	375.44	±	31.31	9.63	±	2.88	UP
34	196.10	±	19.90	8.21	±	1.35	0.03	±	0.00	0.23	±	0.09	3.69	±	0.74	18.76	±	5.09	514.19	±	42.88	2.90	±	0.87	UP
35	120.71	±	12.25	5.69	±	0.94	0.12	±	0.01	0.08	±	0.03	1.00	±	0.20	8.75	±	2.37	154.80	±	12.91	5.26	±	1.57	UP
36	174.71	±	17.73	7.47	±	1.23	0.02	±	0.00	0.16	±	0.06	1.65	±	0.33	8.31	±	2.26	227.90	±	19.01	3.38	±	1.01	UP
37	222.92	±	22.63	10.24	±	1.69	<0.01			0.18	±	0.07	1.95	±	0.39	5.51	±	1.49	219.59	±	18.31	6.39	±	1.91	UP
38	234.35	±	23.79	6.57	±	1.08	0.03	±	0.00	0.18	±	0.02	2.03	±	0.41	10.05	±	2.73	270.16	±	22.53	6.07	±	1.81	UP
40	569.69	±	57.82	7.55	±	1.24	0.08	±	0.01	0.36	±	0.14	5.55	±	1.11	18.49	±	4.02	774.37	±	64.58	5.41	±	1.62	UP
41	251.76	±	25.55	6.73	±	1.11	0.04	±	0.00	0.12	±	0.05	2.13	±	0.43	8.87	±	2.41	318.58	±	26.57	7.89	±	2.36	UP
42	586.22	±	59.50	4.42	±	0.73	<0.01			<0.01			0.34		0.07	13.27	±	3.60	105.10	±	8.77	0.34	±	0.10	UP
43	850.14	±	86.29	5.10	±	0.84	<0.01			<0.01			<0.01			5.44	±	0.48	58.16	±	4.85	<0.01			UP
44	1790.42	±	181.73	6.12	±	1.01	<0.01			0.34	±	0.14	1.02	±	0.20	9.52	±	2.59	179.25	±	14.95	5.44	±	1.63	A
45	2158.68	±	119.11	5.44	±	0.90	<0.01			0.34	±	0.14	1.02	±	0.20	16.67	±	1.53	235.71	±	19.66	2.04	±	0.61	A

Table S5. Plant element concentrations (continuation)

Sample	Al	As	Cd	Co	Cr	Cu	Fe	Li	Land use
46	396.33 ± 125.21	1.24 ± 0.62	<0.01	0.42 ± 0.24	2.86 ± 0.32	6.55 ± 2.58	662.71 ± 164.98	1.66 ± 0.47	A
48	857.50 ± 87.21	0.97 ± 0.16	<0.01	0.59 ± 0.23	2.19 ± 0.44	7.78 ± 2.11	831.50 ± 49.35	1.54 ± 0.46	A
49	387.40 ± 129.54	1.87 ± 0.54	<0.01	0.41 ± 0.12	3.87 ± 0.01	5.87 ± 0.06	533.50 ± 29.70	1.29 ± 0.06	A
50	773.25 ± 327.04	2.45 ± 1.59	<0.01	0.78 ± 0.01	5.31 ± 2.92	7.00 ± 0.43	953.00 ± 62.23	1.76 ± 0.52	A
51	479.85 ± 48.80	0.37 ± 0.06	<0.01	0.42 ± 0.17	5.72 ± 1.15	4.58 ± 1.24	577.50 ± 48.16	1.10 ± 0.33	A
**All soils**									
MIN	70.71	0.37	<0.01	<0.01	<0.01	4.38	58.16	<0.01	
MAX	3086.73	20.25	1.43	1.30	33.38	23.47	1959.97	9.63	
AM	544.95	6.86	0.17	0.29	3.41	9.31	385.96	3.92	
STD	692.31	3.63	0.34	0.28	4.85	4.54	380.22	2.10	
CV	127.04	52.84	204.90	95.95	142.36	48.81	98.51	53.50	
**Agricultural soils (A)**									
MIN	70.71	0.37	<0.01	0.04	0.84	4.58	77.48	1.10	
MAX	3086.73	20.25	1.43	1.30	33.38	23.47	1959.97	7.79	
AM	797.64	7.43	0.33	0.38	4.34	8.53	500.45	3.74	
STD	880.04	4.98	0.52	0.35	6.68	4.25	491.36	1.83	
CV	110.33	66.98	157.00	91.91	153.90	49.82	98.18	49.08	
**Urban park soils (UP)**									
MIN	95.56	3.92	<0.01	<0.01	<0.01	5.44	58.16	0.34	
MAX	1447.57	10.24	0.18	0.36	5.55	18.76	774.37	9.63	
AM	331.43	6.51	0.05	0.18	2.45	10.60	272.11	4.58	
STD	342.39	1.45	0.05	0.08	1.38	4.81	171.66	2.58	
CV	103.31	22.28	94.56	44.89	56.32	45.40	63.08	56.25	
**Industrial park soils (IP)**									
MIN	80.97	5.32	0.02	0.06	1.70	6.13	143.13	2.71	
MAX	360.06	7.04	0.40	0.57	4.41	17.63	688.35	3.90	
AM	2344.44	16.62	0.34	1.30	6.81	10.22	1352.95	5.85	
STD	3086.73	20.25	0.11	0.83	33.38	13.20	1959.97	6.00	
CV	131.66	121.88	32.85	64.01	490.35	129.10	144.87	102.53	

Mean values ± standard deviation; MIN, minimum value; MAX, maximum value; AM, mean values.

Table S5. Plant element concentrations (continuation)

Sample	Mg			Mn			Ni			Pb			Sr			V			Zn			Land use
1	3030.30	±	187.58	34.37	±	9.51	0.86	±	0.06	1.12	±	0.04	381.22	±	35.87	<0.01			22.03	±	3.08	A
2	4343.43	±	268.86	40.65	±	11.24	0.68	±	0.04	1.32	±	0.05	398.04	±	37.46	<0.01			20.21	±	2.83	A
3	2020.20	±	125.05	55.42	±	15.33	2.11	±	0.14	2.72	±	0.10	59.69	±	5.62	<0.01			38.80	±	5.43	IP
4	3333.33	±	206.33	71.76	±	19.85	3.14	±	0.21	1.55	±	0.06	152.72	±	14.37	<0.01			29.41	±	4.12	UP
5	3432.95	±	212.50	38.94	±	10.77	1.23	±	0.08	1.33	±	0.05	295.78	±	27.83	<0.01			24.63	±	3.45	A
6	1515.15	±	93.79	41.08	±	11.36	1.41	±	0.09	1.86	±	0.07	47.05	±	4.43	<0.01			33.82	±	4.73	UP
7	2020.20	±	125.05	59.69	±	16.51	2.20	±	0.14	2.18	±	0.08	108.45	±	10.21	<0.01			35.70	±	4.99	IP
8	2121.21	±	131.30	42.64	±	11.79	0.96	±	0.06	1.47	±	0.05	245.40	±	23.09	<0.01			35.61	±	4.98	UP
9	2222.22	±	137.56	38.95	±	10.77	2.28	±	0.15	1.34	±	0.05	403.03	±	27.93	<0.01			30.75	±	4.30	A
10	1818.18	±	112.55	45.98	±	12.72	1.27	±	0.08	1.33	±	0.05	202.94	±	19.10	<0.01			32.85	±	4.60	UP
11	3232.32	±	200.08	90.10	±	14.92	2.02	±	0.13	1.35	±	0.05	239.48	±	22.54	<0.01			32.10	±	4.49	UP
12	3434.34	±	212.59	40.86	±	11.30	1.26	±	0.08	1.03	±	0.04	139.47	±	13.12	<0.01			52.94	±	7.41	IP
13	3535.35	±	218.84	51.67	±	14.29	1.06	±	0.07	1.71	±	0.06	183.57	±	17.27	<0.01			30.20	±	4.23	A
14	1324.82	±	82.01	47.36	±	13.10	1.83	±	0.12	1.09	±	0.04	127.34	±	11.98	<0.01			29.13	±	4.08	IP
16	2626.26	±	162.57	28.84	±	7.98	1.10	±	0.07	1.30	±	0.05	509.70	±	37.96	<0.01			20.79	±	2.91	A
17	4282.99	±	265.12	37.99	±	10.51	1.22	±	0.08	1.21	±	0.04	457.39	±	43.04	<0.01			12.20	±	1.71	A
18	2727.27	±	168.82	36.56	±	10.11	1.29	±	0.08	1.56	±	0.06	454.51	±	42.77	<0.01			20.51	±	2.87	A
19	7777.78	±	481.44	209.03	±	27.82	0.63	±	0.04	1.25	±	0.05	155.62	±	14.64	<0.01			56.39	±	7.89	UP
20	3939.39	±	243.85	77.48	±	21.43	2.35	±	0.15	1.75	±	0.06	71.80	±	6.76	<0.01			50.10	±	7.01	UP
21	1616.16	±	100.04	40.42	±	11.18	0.70	±	0.05	0.99	±	0.04	20.87	±	1.96	<0.01			29.82	±	4.17	F
22	1707.33	±	105.68	34.07	±	9.42	1.30	±	0.08	0.30	±	0.01	58.54	±	5.51	<0.01			28.26	±	3.95	A
23	4959.14	±	306.97	60.47	±	16.73	3.37	±	0.22	3.00	±	0.11	240.55	±	22.64	1.22	±	0.14	47.55	±	6.65	A
25	2649.16		163.98	28.71		7.94	0.77		0.05	0.84		0.03	479.59		45.13	<0.01			15.03	±	2.10	A
26	3333.33	±	206.33	44.56	±	12.32	3.34	±	0.22	2.03	±	0.07	297.10	±	27.96	<0.01			58.37	±	8.17	A
27	3434.34	±	212.59	39.05	±	10.80	1.97	±	0.13	2.05	±	0.07	249.32	±	23.46	<0.01			87.62	±	12.26	A
28	2828.28	±	175.07	77.05	±	11.31	3.14	±	0.21	17.79	±	0.64	143.03	±	13.46	<0.01			120.80	±	16.90	IP
29	4949.49	±	306.37	70.83	±	19.59	3.62	±	0.24	4.27	±	0.15	230.51	±	21.69	1.94	±	0.22	125.91	±	17.62	A
30	2675.16	±	165.59	66.82	±	18.48	10.57	±	0.69	7.61	±	0.28	164.16	±	15.45	2.89	±	0.32	95.10	±	13.31	A
31	2424.24	±	150.06	56.58	±	15.65	1.12	±	0.07	3.16	±	0.11	136.33	±	12.83	<0.01			64.59	±	9.04	A
32	3434.34	±	212.59	44.36	±	12.27	1.62	±	0.11	1.84	±	0.07	153.37	±	14.43	<0.01			31.08	±	4.35	UP
33	1564.87	±	96.87	44.57	±	12.33	1.07	±	0.07	1.78	±	0.06	156.17	±	14.70	<0.01			32.45	±	4.54	UP
34	5555.56	±	343.89	67.20	±	18.59	2.51	±	0.16	2.60	±	0.09	323.10	±	30.40	<0.01			41.09	±	5.75	UP
35	3737.37	±	231.34	40.09	±	11.09	2.01	±	0.13	1.94	±	0.07	164.48	±	15.48	<0.01			27.61	±	3.86	UP
36	3737.37	±	231.34	46.21	±	12.78	3.29	±	0.22	2.97	±	0.11	354.81	±	33.39	<0.01			34.07	±	4.77	UP
37	5151.52	±	318.88	50.97	±	14.10	2.47	±	0.16	2.28	±	0.08	103.20	±	54.99	<0.01			23.20	±	3.25	UP
38	4040.40	±	250.10	40.48	±	11.20	1.70	±	0.11	1.62	±	0.06	130.68	±	12.30	<0.01			28.61	±	4.00	UP
40	1919.19	±	118.80	45.37	±	12.55	1.89	±	0.12	4.08	±	0.15	174.92	±	16.46	<0.01			59.92	±	7.38	UP
41	4505.57	±	278.89	45.38	±	12.55	1.27	±	0.08	2.17	±	0.08	167.77	±	15.79	<0.01			45.64	±	6.39	UP
42	4520.07	±	279.79	37.07	±	10.25	1.36	±	0.09	0.68	±	0.02	200.00	±	18.82	<0.01			19.73	±	2.76	UP
43	2968.03		183.72	25.51	±	7.06	0.68	±	0.04	0.34	±	0.01	97.62	±	9.19	<0.01			22.45	±	3.14	UP
44	2291.16	±	141.82	22.45	±	6.21	2.04	±	0.13	1.02	±	0.04	478.57	±	45.03	<0.01			19.73	±	2.76	A
45	2662.93	±	164.84	57.48	±	15.90	1.36	±	0.09	1.02	±	0.04	82.31	±	7.75	<0.01			79.93	±	9.18	A
46	1759.63	±	542.21	48.11	±	8.22	1.61	±	0.13	0.78	±	0.12	31.45	±	7.74	0.74	±	0.07	11.20	±	3.08	A
48	1975.50	±	122.28	209.70	±	38.00	1.23	±	0.08	1.65	±	0.06	55.20	±	5.19	1.76	±	0.20	22.86	±	3.20	A

Table S5. Plant element concentrations (continuation)

Sample	Mg	Mn	Ni	Pb	Sr	V	Zn	Land use
49	2346.50 ± 247.49	109.00 ± 49.29	1.47 ± 0.25	1.78 ± 0.06	46.20 ± 17.25	0.86 ± 0.22	15.60 ± 3.17	A
50	2231.25 ± 384.31	234.10 ± 34.75	1.82 ± 0.63	1.11 ± 0.13	49.81 ± 13.78	1.79 ± 0.37	10.20 ± 2.62	A
51	1855.50 ± 114.86	62.55 ± 17.30	2.09 ± 0.14	0.59 ± 0.02	78.75 ± 7.41	0.85 ± 0.09	19.87 ± 2.78	A
				**All soils**				
MIN	1324.82	22.45	0.63	0.30	20.87	<0.01	10.20	
MAX	7777.78	234.10	10.57	17.79	509.70	2.89	125.91	
AM	3097.26	60.39	1.92	2.14	202.16	1.51	38.86	
STD	1289.04	44.85	1.51	2.63	136.73	0.73	26.18	
CV	41.62	74.27	78.66	122.48	67.63	48.80	67.37	
				**Agricultural soils (A)**				
MIN	1707.33	22.45	0.68	0.30	31.45	<0.01	10.20	
MAX	4959.14	234.10	10.57	7.61	509.70	2.89	125.91	
AM	2932.85	63.15	2.06	1.83	250.51	1.51	38.40	
STD	961.60	53.49	2.03	1.54	166.61	0.73	31.66	
CV	32.79	84.71	98.40	84.34	66.51	48.80	82.46	
				**Urban park soils (UP)**				
MIN	1515.15	25.51	0.63	0.34	47.05		19.73	
MAX	7777.78	209.03	3.29	4.08	354.81		59.92	
AM	3603.98	59.18	1.76	1.83	174.51	<0.01	35.34	
STD	1590.54	40.68	0.77	0.84	79.25		11.24	
CV	44.13	68.73	43.98	45.82	45.41		31.82	
				**Industrial park soils (IP)**				
MIN	1324.82	40.86	1.26	1.03	59.69		29.13	
MAX	3434.34	77.05	3.14	17.79	143.03		120.80	
AM	4949.49	70.83	3.62	4.27	230.51	<0.01	125.91	
STD	3434.34	66.82	10.57	7.61	164.16		95.10	
CV	69.39	94.35	292.19	178.14	71.22		75.53	

Mean values ± standard deviation; MIN, minimum value; MAX, maximum value; AM, mean values.
